# Contextual factors related to aging determine force-based manipulation dosage: a prospective cross-sectional study

**DOI:** 10.1186/s12998-025-00584-1

**Published:** 2025-05-21

**Authors:** Michele J. Maiers, Alexander R. Sundin, Ryan J. Oster, Steven Kreul, Quinn Malone, Steven R. Passmore

**Affiliations:** 1 Northwestern Health Sciences University, Bloomington, MN USA; 2 RAND Research Across Complementary and Integrative Health Institutions (REACH) Center, Santa Monica, CA USA; 3 School of Health and Exercise Sciences, University of British Columbia: Okanagan, Kelowna, BC Canada; 4 Faculty of Kinesiology and Recreation Management, University of Manitoba, Winnipeg, MB Canada

**Keywords:** Force-based manipulation, Spinal manipulation, Chiropractic, Kinetic and kinematic characteristics, Ageing, Older adults, Contextual factors

## Abstract

**Background:**

Contextual factors influence clinicians’ delivery of force-based manipulation (FBM), like spinal manipulative therapy (SMT). It is particularly important to discern how contextual factors interact with therapeutic forces delivered to an older adult population, to minimize risk and identify ideal dosage. This study aimed to determine whether contextual factors pertaining to aging result in the modulation of kinetic and kinematic parameters used by experienced clinicians when delivering SMT.

**Methods:**

Participants were randomly presented with a series of 12 AI-generated patient vignettes, featuring both visual and auditory content and representing varying age-related contextual factors. Factors included chronological (35-, 65- and 85-year-old), pathological (“healthy” vs degenerative spine), and felt (perceived as “young” vs. “old”) age. Participants delivered SMT to a human analogue manikin based on each vignette, presented six times in randomized order. Kinetic and kinematic parameters were collected and analyzed for differences between “young” and “old” contextual factors of age, using a 3-way repeated measures ANOVA model.

**Results:**

Sixteen licensed chiropractors (8 female, 8 male) participated, with an average age of 45.4 (SD = 9.7, range 34–64) years and 18.3 (SD = 10.8, range 5–39) years of experience. A main effect in peak force was found for both chronological (F(_2,30_) = 26.18; *p* <.001, η_p_^2^ = 0.636) and pathological age (F(_1,15_) = 11.58; *p* =.004, η_p_^2^ = 0.436), following a stepwise progression of decreased force with increased age and with pathology. No statistically significant differences were found in peak force based on felt age, or in time to peak force for any factor. A main effect was found for chronological age with peak acceleration (F(_2,20_) = 9.50; *p* <.001, η_p_^2^ = 0.487) and peak velocity (F(_2,20_) = 7.20; *p* =.004, η_p_^2^ = 0.419), but not for pathological or felt age. There was a significant difference in time to peak velocity for felt age (F(_1,10_) = 12.23; *p* =.006, η_p_^2^ = 0.550), with a shorter time to peak velocity in response to vignettes with older felt age.

**Conclusion:**

Contextual factors of aging modulated certain kinetic and kinematic characteristics when delivering SMT. This provides evidence that practitioners differentially discern aspects of aging to inform how they deliver FBM dosage. Future research is needed to identify ideal kinetic and kinematic characteristics based on considerations of aging.

**Supplementary Information:**

The online version contains supplementary material available at 10.1186/s12998-025-00584-1.

## Introduction

Contextual factors of aging may be important considerations for force-based manipulation (FBM), including dosage of spinal manipulative therapy (SMT). Given the growth of the aging segment of the population worldwide, safe and effective treatments to maintain spinal health and decrease spine-related disability are increasingly important. Spinal health is central to healthy aging, as it facilitates physical function, quality of life, and aging in place. However, spinal pain is common in old age [1], and older adults report more severe disability due to back pain than younger adults [2, 3]. Back pain is identified as one of the leading causes of disability in adults 60 years of age and older, resulting in substantial social and economic loss [3–5].

Clinical practice guidelines and systematic reviews recommend SMT as a first-line intervention for back pain, including for older adults [6–9]. As a FBM, SMT is associated with a heightened risk of adverse events among older adults due to age-related factors, including frailty, degenerative changes, and bone density [10, 11]. Treatment consideration for appropriate dosage for older adults is thus warranted [12]. Few randomized controlled trials investigating the use of FBM in an older adult population have reported data on adverse events [6]. Therefore, guidance about FBM dose risk is inadequate. Excessive force magnitudes are suggested contributors to adverse events following thoracic SMT delivered to the older adult population, specifically rib fracture [13, 14]. Therefore, understanding practitioners’ modulation of output when delivering SMT to older adults is an important step toward identifying the safest and most effective dose ranges applied to this population.

Limited research suggests that clinicians modulate technique and force when delivering SMT based on individual patient contextual factors, including age, apparent sex, stature, degenerative change, and pathology [15, 16]. The degree to which these characteristics influence clinician force output is poorly understood and may be especially nuanced in an older adult population for whom the contextual factors of chronological, pathological, and felt age are varied. Limited research has examined the kinetic and kinematic of SMT delivered to older adults. One study investigating kinetic characteristics of SMT delivered to individuals ≥ 65 years of age found data to be comparable to the values reported in the literature for younger adults [17]. Their study was limited in that it relied on a single practitioner and did not consider age-related contextual variations, such as degenerative changes of the spine, frailty, or perception of age among the sample [17]. A more recent study found significant differences in the peak force and thrust speed of SMT delivered by chiropractors viewing case vignettes of a 30-, 50-, and 70-year-old patient [18]. Only chronological age was considered as a factor, and advanced old age was not considered [18].

Though there is evidence that SMT force-based parameters are modulated by clinicians based on the chronological age of the patient, it is unknown whether spinal manipulation forces delivered to older adults are modulated by age-based contextual factors like degeneration, fragility, or vibrancy. The purpose of this study was to elucidate whether contextual factors pertaining to aging, including chronological, pathological, and felt age, result in the modulation of kinetic and kinematic parameters used by clinicians delivering SMT. Our hypothesis was that the presentation of vignettes of patients appearing to be older in chronological, pathological, and felt age would influence FBM dosage.

## Methods

This experimental cross-sectional study examined force-based parameters used by experienced chiropractors delivering SMT. It was conducted at Northwestern Health Sciences University (NWHSU) in Bloomington, Minnesota, USA and approved by the NWHSU Institutional Review Board. Data was collected between September 1 and October 31, 2024.

### Study participants

Participants included English-speaking, licensed Doctors of Chiropractic with at least five years of clinical experience. Additional inclusion criteria included a current active clinical practice of at least 20 h/week; self-reported utilization of high-velocity low amplitude (HVLA) spinal manipulative therapy (SMT) on at least 50% of patients; and self-reported patient base consisting of at least 10% adults aged 65 years and older. Exclusion criteria included practitioners who perceived themselves as physically unable to deliver 72 manipulative thrusts to a manikin in 90 min, and those unfamiliar with performing prone thoracic region SMT delivered as a bilateral thenar transverse push [19]. Participants were compensated with a $50 gift card after participating in the study.

Recruitment of participants was conducted through email, social media, and organizational newsletters sent by state and regional chiropractic professional associations, in addition to NWHSU’s alumni and clinical networks. Interested individuals responded to recruitment materials via a website address or QR code, which led to a website that included a description of the study, risks and benefits of participating, and an automated online survey to determine eligibility.

### Study design

Participants were first fitted with an accelerometer (see below) and oriented to a human analogue manikin (HAM®, Canadian Memorial Chiropractic College, Toronto, ON, Canada) upon which they would be delivering an HVLA thrust. They were allowed to practice the procedure on the manikin until they felt comfortable performing it. Participants were then told they would see and hear a series of patient vignettes, all of whom were safe to adjust with no contraindications to SMT. To avoid introducing bias, there was no pre-briefing discussion of differences between the vignettes or contextual factors associated with aging. After each case was presented, participants were instructed to deliver a thrust to the manikin when they felt ready to do so, modifying their adjustment based on the patient vignette they had just seen and heard. There were no time restrictions on when the thrust was delivered relative to seeing and hearing the vignette; audio cues were played only once.

After this orientation, participants were presented with the series of AI-generated mock audiovisual patient vignettes representing varying age-related contextual factors, including chronological, pathological, and felt age. After seeing and hearing text, visual, and audio cues for each vignette (see Patient Vignettes, below), participants delivered a thrust to the manikin. All possible permutations of the age-related contextual factor combinations were presented, with each permutation represented by a single patient for a total of 12 cases. Each case was presented six times each in a randomized fashion to prevent possible order effects, for a total of 72 trials.

### Patient vignettes

Mock patient vignettes were projected onto a screen in a randomized order using E-Prime® 2.0 (Psychology Software Tools, Sharpsburg, Pennsylvania). Vignettes were based on a common presenting complaint of uncomplicated mid-thoracic spine pain, rated 5/10 on a visual analog scale, lasting three months in duration. Cases were further modified to represent 12 variations on three contextual factors of aging, described below.

Open AI was used to create images for each of the 12 mock patients. Radiographs of the thoracic spine (Radiopedia stock images, accessed Sept. 2, 2024) were displayed alongside a patient image and written description of their complaint. Contextually appropriate audio simulating the voice of mock patients briefly describing their back-related health was generated by Murf.ai (Salt Lake City, Utah, USA) and presented simultaneously with visual stimuli. The mock patient vignettes used to represent differences in chronological, pathological, and felt age can be found in supplementary material.

Chronological age is the number of years lived by an individual and was represented in this study by the stated age in the written description of mock patients who were either 35, 65, or 85 years old. The images of mock patients were designed to visually approximate stereotypes for those ages, including hair color and skin wrinkling.

Pathological age was defined as chronological age plus a deviation based on the progression of a degenerative condition [20]. In this case, the presence or absence of spinal degeneration was used to represent either a “young” spine (i.e., adequate bone mass and no degenerative changes) or an “old” spine (i.e., evidence of osteopenia, degenerative disc and joint disease). Pathological age was represented in the vignettes by images of anterior-to-posterior and lateral thoracic spine radiographs.

Felt age was defined as the perceived age of a person based on appearance and behavior. It can be described as how old or young a person feels, or is perceived to be, relative to their chronological age [21]. Felt age was represented in mock patient vignettes by a brief recorded verbal narrative played alongside the visual case description. Each vignette had a unique, AI-generated age-appropriate voice. Two scripts were used. One was meant to represent “young” felt age: “Even though I have back pain, I feel really good for my age.” A second represented “old” felt age: “Especially with this back pain, I’m really starting to feel my age.”

### Data collection and analysis

**Fig. 1:**
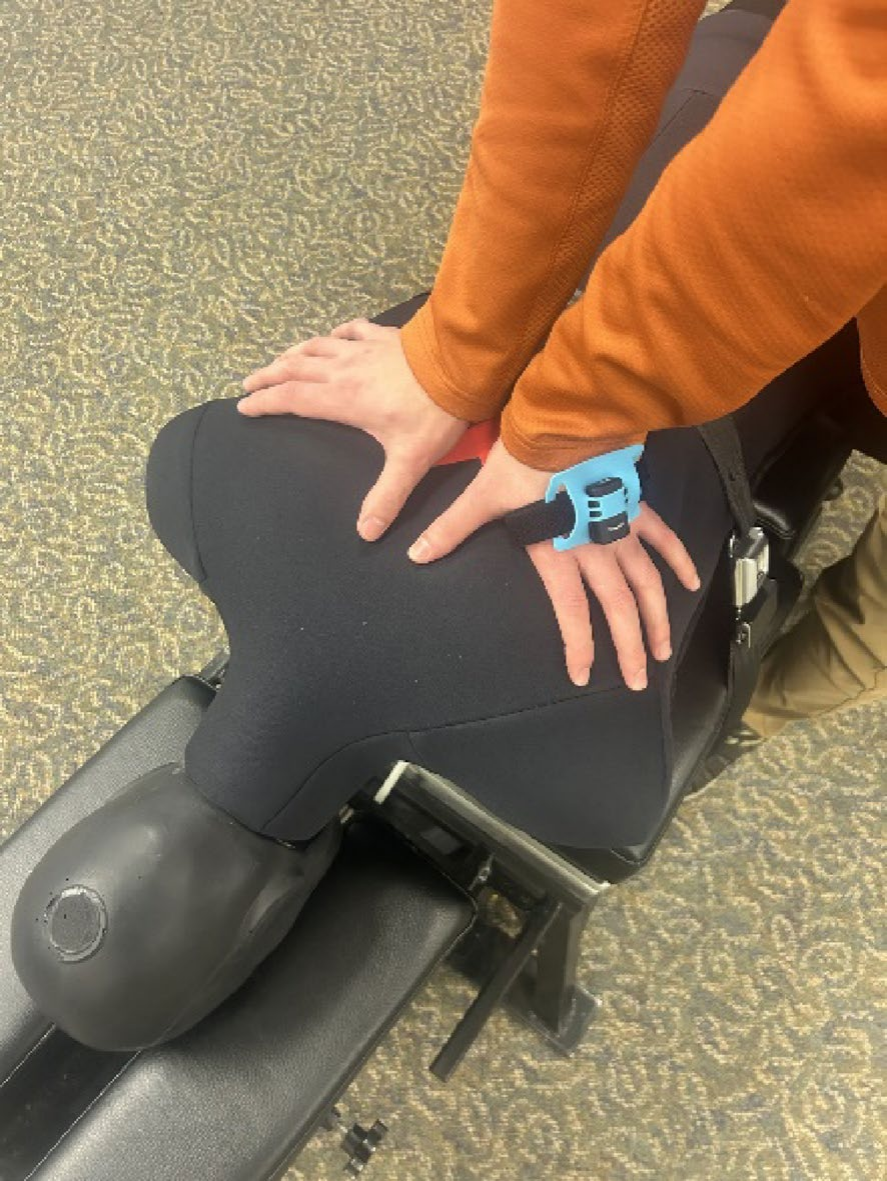
Participant hand and accelerometer placement to deliver SMT to the HAM®, on the FSTT®

Participants were instructed to perform a bilateral thenar transverse push, an HVLA SMT maneuver common to clinical practice (see Fig. 1) [19], based on each case vignette presented. The manipulation was applied to an “X” located on the mid-thoracic region of a prone human analogue manikin (HAM®) in response to each patient vignette. Participants were instructed to “adjust this patient as you would in practice.” The manikin was strapped to an FSTT® (Canadian Memorial Chiropractic College, Toronto, ON, Canada) treatment table, instrumented to collect three-dimensional forces located at a force plate to table interface [22]. The FSTT® comprises a Leander 900 Z Series treatment table (Leander Health Technologies Corporation, Lawrence, Kansas, MO) instrumented with an embedded force plate sampling at 1000 Hz in the thoracic portion (OR6-7; Advanced Mechanical Technology Inc, Watertown, MA). All relevant details concerning the precision of the embedded force plate may be found on the manufacturer's website in the manual available for download [23]. Calibration was performed by the manufacturer. The manikin torso was positioned over the aspect of the table intended to support and record manipulation of the thoracic region. Force sensing tables, and the biomechanical variables measured from them, are established and widely accepted in determining spinal manipulation force loads and timing [24]. Preload force (the force held prior to impulse thrust, in N), peak force (the greatest force during the manipulative thrust, in N), and time to peak force (time from the end of the preload force to peak force, in ms) were measured. Similar to other research, each of the aforementioned force variables were mathematically defined and determined by the standardized algorithms of the FSTT® software [25].

To capture output from the clinician to manikin interface, participants were fitted with wireless inertial motion units (IMUs) (3-Space™ Mini Bluetooth, Yost Labs Inc., Portsmouth, OH. USA) positioned over the dorsal aspect of the third metacarpal of the left hand, pointing distally [26]. All relevant details concerning the precision of the IMUs may be found on the manufacturer's website [27]. This device was designed for autocalibration by the manufacturer. Acceleration data was captured at 50 Hz via the IMUs. The ‘linear acceleration’ datasets from the IMUs were used for all outcomes. This data represents the acceleration converted into global space using Yost Lab’s orientation estimation filters, with gravity and magnetic north as the reference axes for global space. This effectively means that each acceleration axis did not move with the accelerometer but remained fixed along a constant line, representing an unmoving global reference frame. Additionally, the effects of gravity were accounted for and were removed from the produced acceleration measurements.

Before any analysis, data for each acceleration axis were filtered with a 4th order, zero-lag, high-pass Butterworth filter with a critical frequency of 0.5 Hz. The derivative of the resultant acceleration was calculated to obtain jerk, while each filtered acceleration axis was integrated to obtain velocity. Each velocity axis was further filtered using the same method noted above, and the resultant velocity calculated from the filtered axes. For outcomes gained from the IMU measurements, the start of the SMT was defined as the time in the last 4 s of recording at which jerk exceeded 2 standard deviations from the mean jerk for at least 2 samples (40 ms). While the mean and standard deviation of jerk were calculated from the whole trial recording, only the last 4 s of each trial recording were considered for the determination of movement onset. For the calculation of acceleration and velocity peaks, the resultant filtered data were used. As some participants quickly released the manikin after performing their spinal manipulation in some trials, time-to-peak values were defined as the time from movement onset to the peak value within 500 ms after movement onset. Reported outcomes include peak acceleration (m/s^2^), peak velocity (m/s), time to peak acceleration (s) and time to peak velocity (s).

Microsoft Copilot AI was used to generate the code required to identify peak acceleration values from raw accelerometer data. The code was applied, and an analysis script was generated for MATLAB® (Mathworks, Natick, MA). The researchers (QM and SP) reviewed the MATLAB® script and performed manual edits to output code as needed. FSTT and accelerometer data were collected independently with temporal overlap but not triggered in synchrony, since they were considered separately and independently in analyses and not directly compared.

### Statistical models

Sample size was calculated based on three-way analysis of variance (ANOVA). A-priori power calculations were conducted using G*Power (Version 3.1.9.6) based on conservative effect size estimates from a comparable study [28]. To attain a power of β = 0.95 and significance ɑ ≤ 0.05, 12 participants were required. To account for possible invalid trials due to technical issues, a total of 16 participants were recruited.

Descriptive statistics were used to describe clinician participants. Separate repeated measures ANOVA models were applied to the data for each kinetic or kinematic variable. For each variable, the mean of each series of six trials was used in analysis, excluding outliers (values > 2.5 standard deviations from the mean) or trials lost due to equipment malfunctions. Q–Q plots were inspected to ensure normality. Sphericity of the data was assessed using Mauchly’s test; no corrections were needed. Standard error of the mean was calculated to understand the precision of the sample mean relative to a true population [29]. Tukey’s Honestly Significant Differences were used as post-hoc tests to determine specific differences for all main effects or interactions involving more than two levels.

## Results

Sixteen licensed chiropractors participated, 8 of whom self-identified as female and 8 identified as male. The average age of participants was 45.4 (SD = 9.7, range 34–64) years. Participants reported delivering patient care an average of 31 (SD = 5.7, range 20–40) hours per week; mean years in practice was 18.3 (SD = 10.8, range 5–39).

Of 1152 SMT trials recorded, force data from 1003 were included in the analysis; 149 (12.9%) were identified as erroneous due to equipment or software errors. An additional 27 data points were excluded as outliers, resulting in the removal of less than 1% of data points. Technical issues were discovered with the accelerometer data from five participants and were subsequently excluded, leaving data from 11 participants for that analysis.

**Fig. 2:**
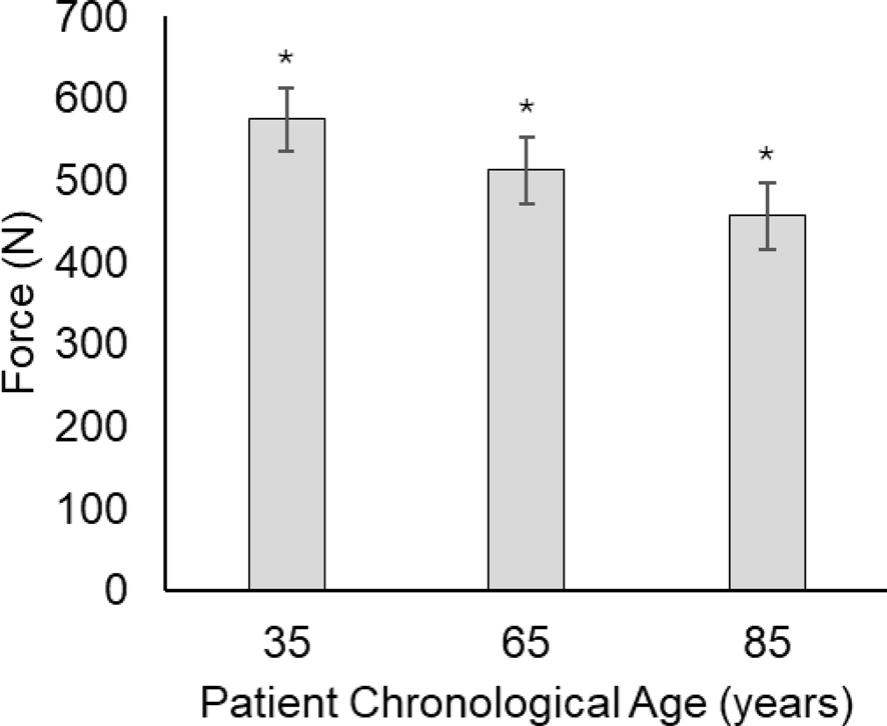
Main effect in peak force for chronological age. *Denotes significant difference compared to all other data levels (*p* ≤ 0.05)

For peak force, a main effect was found for chronological age (F(_2,30_) = 26.18; *p* < 0.001, η_p_^2^ = 0.636), following a stepwise progression of significantly decreased force with each increase in chronological age (see Table 1 and Fig. 2). There was also a significant difference between peak forces based on pathological age (F(_1,15_) = 11.58; *p* = 0.004, η_p_^2^ = 0.436), with greater force used when the vignette presented a younger pathological age (see Table 2). There was no significant difference between peak forces based on felt age (see Table 3), and no differences in time to peak force for any factor.

**Table 1:** Kinetic and kinematic parameters of SMT based on chronological age

	Chronological age (years)		
	35	65	85
Preload force (N; m*ean* ± *SE*) F(_2,30_) = 2.05; *p* =.147, η_p_^2^ = 0.120	115 ± 11.6	116 ± 12.3	109 ± 10.6
Peak force (N; *mean* ± *SE*)* F(_2,30_) = 26.18; *p* <.001, η_p_^2^ = 0.636	575 ± 37.8^†^	513 ± 40.5^†^	457 ± 41.3^†^
Time to peak force (ms; *mean* ± *SE*) F(_2,30_) = 2.79; *p* =.077, η_p_^2^ = 0.157	139 ± 7.3	137 ± 7.1	134 ± 6.3
Peak acceleration (m/s^2^; *mean* + *SE*)* F(_2,20_) = 9.50; *p* <.001, η_p_^2^ = 0.487	8.44 ± 1.83^†^	7.42 ± 1.80	6.79 ± 1.78
Peak velocity (m/s; *mean* + *SE*)* F(_2,20_) = 7.20; *p* =.004, η_p_^2^ = 0.419	0.31 ± 0.05^†^	0.27 ± 0.05	0.25 ± 0.06
Time to peak acceleration (s; *mean* + *SE*) F(_2,20_) = 0.70; *p* =.507, η_p_^2^ = 0.066	0.12 ± 0.01	0.12 ± 0.01	0.12 ± 0.01
Time to peak velocity (s; *mean* + *SE*) F(_2,20_) = 0.46; *p* =.638, η_p_^2^ = 0.044	0.15 ± 0.01	0.16 ± 0.01	0.16 ± 0.01

F-Statistics and associated *p* values reflect main effects

*Denotes significant difference (*p* < 0.05); N = newton; SE = standard error; ms = millisecond; m = meter; s = second

^†^Denotes significant post-hoc differences in pair-wise comparisons based on Tukey’s Honestly Significant Difference tests

**Table 2:** Kinetic and kinematic parameters of SMT based on pathological age

	Pathological age	
	Young	Old
Preload force (N; *mean* ± *SE*) F(_1,15_) = 0.02; *p* =.882, η_p_^2^ = 0.002	114 ± 11.1	113 ± 11.6
Peak force (N; *mean* ± *SE*)* F(_1,15_) = 11.58; *p* =.004, η_p_^2^ = 0.436	526 ± 39.1	504 ± 38.7
Time to peak force (ms; *mean* ± *SE*) F(_1,15_) = 079; *p* =.389, η_p_^2^ = 0.050	137 ± 7.0	136 ± 6.7
Peak Acceleration (m/s2; *mean* + *SE*) F(_1,10_) = 0.008; *p* =.977, η_p_^2^ = 0.000	7.56 ± 1.76	7.55 ± 1.83
Peak Velocity (m/s; *mean* + *SE*) F(_1,10_) = 0.06; *p* =.814, η_p_^2^ = 0.006	0.28 ± 0.05	0.28 ± 0.05
Time to peak acceleration (s; *mean* + *SE*) F(_1,10_) = 0.09; *p* =.772, η_p_^2^ = 0.009	0.12 ± 0.01	0.12 ± 0.01
Time to peak velocity (s; *mean* + *SE*) F(_1,10_) = 2.81; *p* =.125, η_p_^2^ = 0.219	0.16 ± 0.01	0.15 ± 0.01

F-Statistics and associated *p* values reflect main effects

*Denotes significance (*p* ≤ 0.05); N = newton; SE = standard error; ms = millisecond; m = meter; s = second

**Table 3:** Kinetic and kinematic parameters of SMT based on felt age

	Felt age	
	Young	Old
Preload force (N; *mean* ± *SE* F(_1,15_) = 0.02; *p* =.898, η_p_^2^ = 0.001	113 ± 11.9	114 ± 10.9
Peak force (N; *mean* ± *SE*) F(_1,15_) = 1.74; *p* =.207, η_p_^2^ = 0.104	509 ± 38.1	520 ± 39.8
Time to peak force (ms; *mean* ± *SE*) F(_1,15_) = 0.10; *p* =.759, η_p_^2^ = 0.006	137 ± 6.83	137 ± 6.84
Peak Acceleration (m/s^2^; *mean* + *SE*) F(_1,10_) = 0.13; *p* =.727, η_p_^2^ = 0.013	7.54 ± 1.78	7.57 ± 1.80
Peak Velocity (m/s; *mean* + *SE*) F(_1,10_) = 1.85; *p* =.204, η_p_^2^ = 0.156	0.28 ± 0.06	0.27 ± 0.05
Time to peak acceleration (s; *mean* + *SE*) F(_1,10_) = 1.86; *p* =.203, η_p_^2^ = 0.157	0.12 ± 0.01	0.12 ± 0.01
Time to peak velocity (s; *mean* + *SE*)* F(_1,10_) = 12.23; *p* =.006, η_p_^2^ = 0.550	0.17 ± 0.01	0.15 ± 0.01

F-Statistics and associated *p* values reflect main effects

*Denotes significance (*p* ≤ 0.05); N = newton; SE = standard error; ms = millisecond; m = meter; s = second

Post-hoc between-subject factor analyses were conducted based on the gender of clinician participants, eight of whom identified as female and eight as male. For peak force, the resulting mixed factorial ANOVA yielded a significant between participant main effect (F(_1,14_) = 4.95; p = 0.043, η_p_^2^ = 0.261). Overall, female clinicians (M = 438 N, SE = 48.8) thrusted with lower peak force than male clinicians (M = 591 N, SE = 48.8). There were no significant between gender differences in preload force or time to peak force.

An additional post-hoc between-subject factor analysis based on years in practice (5–19 versus > 20 years) showed no significant between-group differences.

For peak acceleration, a main effect was found for chronological age (F(_2,20_) = 9.50; *p* < 0.001, η_p_^2^ = 0.487). Post-hoc analysis revealed that participants produced greater peak acceleration when responding to the 35-year-old patient vignettes compared to all other ages (see Table 1), although post-hoc analyses demonstrated no difference between the 65- and 85-year-olds. There were no statistically significant main effects for peak acceleration based on pathological or felt age (see Tables 2 and 3).

For peak velocity, a main effect was found for chronological age (F(_2,20_) = 7.20; *p* = 0.004, η_p_^2^ = 0.419). Like peak acceleration, post-hoc analyses revealed that peak velocities were greater when participants were responding to the 35-year-old patient vignettes compared to both other ages (see Table 1), but without significant difference between peak velocities when responding to the 65- and 85-year-old vignettes. There were no statistically significant main effects for peak velocity based on pathological or felt age (see Tables 2 and 3).

There were no significant differences in time to peak acceleration for any factor. There was a significant difference in time to peak velocity for felt age (F(_1,10_) = 12.23; *p* = 0.006, η_p_^2^ = 0.550). Participants took less time to reach their peak velocity when responding to vignettes representing a population who felt older (M = 0.15 s, SE = 0.01) compared to younger (M = 0.17 s, SE = 0.01) (see Table 3).

## Discussion

Clinicians in this study modulated kinetics and kinematic parameters when delivering SMT based on varied contextual factors of aging. Modifications included a decrease in peak force with increased chronological and pathological age; increase in peak acceleration and peak velocity with the youngest chronological age; and decreased time to peak velocity with increased felt age. While modulation was observed in response to the aforementioned contextual factors of aging, this was not a consistent finding across all factors and for all kinetic and kinematic variables considered. Our findings are consistent with previous research demonstrating decreased peak force used on patient vignettes with older chronological age, and expands on that finding to include advanced old age [18]. As a mechanical intervention, clinicians delivering FBM may be most responsive to the contextual factors of aging likely to impact the physical properties of the area their force is applied to. This contrasts with felt age, which is more psychosocial in nature [21]. While not all contextual factors associated with aging appeared to influence SMT delivery, they remain important considerations for a person-centered, bio-psycho-social approach to care overall. To our knowledge, this is the first study examining SMT dosage applied to the older adult spine that considered varied contextual aspects of aging in a controlled manner.

Neither peak preload force nor time to peak force were influenced by any of the three examined contextual aspects of aging. This provides evidence that clinicians apply consistent preload stress and temporal parameters across differently aged patients during SMT. Peak acceleration was greater for the youngest chronological age. The modulation of thrust force and acceleration appears independent of peak preload force and time to peak acceleration, consistent with another study of SMT in younger adults [26].

We also found a main effect indicating that female participants were more conservative in their peak force delivery than males. This difference in gender was consistent with previous studies involving trainees [30, 31], but contrasts with another study involving experienced clinicians [32]. Differences may have occurred in findings due to the technique required in the thoracic spine SMT delivery. The current study required a prone, bilateral thenar push, while the previous study using experienced clinicians allowed for any technique with a posterior to anterior thrust. Additionally, the present study utilized manikins, while the previous study utilized in vivo participants as SMT recipients, which may have altered clinician to force target interfaces and dispersion [32].

Like other studies using the FSTT® [18, 25], we observed considerable between-provider variability. Some participants’ greatest force output was less than the lightest force output of others. However, participants demonstrated modulation of force components in response to the differing contextual factors of chronological age and pathological age regardless of the amount of force applied. Therefore, variability in between-participant comparisons should be expected, and may be only important if optimized ranges for peak force can be identified as therapeutic targets. To date, therapeutic force targets have not been established. Most participants in this study learned SMT prior to the routine use of technology-driven force–time feedback in technique laboratories. As a result, they were not taught to target specific force values based on numeric feedback, but based upon clinical outcomes or verbal feedback from instructors or patients. As force-sensing technology becomes common in educating clinicians who deliver FBM, the clinical relevance of targeted force ranges will necessitate further study to ensure these values are based on empirical evidence. The results of this study suggest that clinicians feel the need to vary their SMT dosage depending on patient characteristics, likely to achieve desired clinical outcomes and avoid harm. Considering the variability of kinetic and kinematic outcomes between individual participants, and the demonstrated variation of these values based on patient characteristics, clinically standardized peak force ranges for FBM will need to vary depending on patient characteristics.

Older adults’ musculoskeletal systems change with age, including reduced muscle mass and joint degeneration [33]. While these changes are an expected part of the aging process, age may present differently based on contextual aspects of aging. This includes the age at which degeneration occurs, the degree to which comorbidities contribute to the pathological age of the spine, and how these changes impact an individual’s perception of their felt age. Assessment of not just chronological age, but also pathological and felt age, is necessary for thorough assessment and care planning for older adults with spine-related pain and disability.

While quantifying kinetic characteristics during SMT has been identified as a seminal next-step priority in understanding the components responsible for clinical effectiveness and delivery [34], exploring kinematic parameters are also important. Kinematic parameters allow an understanding of how a clinician plans the execution of their movement. The results that demonstrate modulation of peak acceleration and peak velocity in this study complemented force outcomes related to chronological age. A shorter time to peak velocity with older felt age suggests that clinicians used that information to preplan their movements and ballistically deliver their dose [35].

Understanding and defining the dose of a FBM procedure is important for three reasons: first, to ensure that the patient is receiving enough of the intervention to be therapeutically useful; second, to ensure that the patient is not receiving too much of the intervention, or an “overdose”, which could leave to an adverse event; and third, so that clinicians delivering SMT can be trained in best practices, learning the appropriate parameters of motor skill acquisition and performance to deliver clinically therapeutic FBM without leading to an overdose. This study provides foundational data for this line of inquiry, reporting mean and ranges of kinetic and kinematic parameters delivered by 16 experienced clinicians. We have demonstrated that clinicians modulate their force based on both the chronological and pathological age of patients. This knowledge will inform future studies which should examine the safety of SMT delivered to older adults, targeted dosing to deliver an optimized therapeutic effect, and standardized education for providers delivering SMT in the growing older adult population.

### Limitations

Similar to other studies collecting kinetic and kinematic data, some force (13%) and accelerometer (31%) data were not included in the present analyses due to errors related to the data capture technology. To mitigate the impact of these errors, we enrolled more participants than needed based on our sample size calculation.

Since SMT occurs over a relatively short time (approximately 500 ms), sampling at a high enough rate is crucial to capture peak kinetic, kinematic, and temporal values. The sampling rate at which IMU data were collected (50 Hz) may be nearly too low to capture movement parameters of SMT accurately. Thus, the acceleration and acceleration-derived values reported presently may be more variable than those derived from data which was sampled at a higher rate due to peak attenuation and potential aliasing. Future studies should consider sampling at a higher rate than we used to avoid these potential issues.

Artificial Intelligence (AI) was used to depict contextual aspects of aging among the mock patient vignettes used in this study. AI is widely understood to be an evolving technology and may not have adequately captured the intended contrast between contextual factors of aging tested here. Further, the researchers’ (MM, AS, SP) own biases about aging may have inappropriately influenced the representation of contextual factors across vignettes. Perception of contextual factors is subjective, and thus may not be consistently recognized or interpreted across providers, potentially confounding the generalization of aging concepts. Moreover, it is possible that clinician participants in this study did not have adequate time to digest contextual factors to the same extent as might be obtained during a clinical encounter with live conversation, observed movements, and behaviors. Future research may include qualitative studies to identify how clinicians consciously perceive the stereotypical audiovisual aspects of the contextual factors of aging that were studied here, and implement this information into AI-generated vignettes.

When generating mock patient vignettes, we attempted to eliminate gender as a variable to try to focus participants’ attention on aspects of aging. Other research suggests that patient gender can influence clinicians’ force output [16]. This may be especially true for older adult patients, given the prevalence of osteoporosis is greater among women compared to males [36]. We did not assess whether our effort to create gender-neutral vignettes was effective for the intended purpose.

To isolate changes in contextual factors of interest, we attempted to standardize other factors that may influence SMT delivery. In this study, all participants delivered SMT on the same human analog manikin (HAM®). The practice of using the HAM® as an SMT target is common in literature pertaining to SMT force delivery [18, 25]. While a standardized tactile presentation may have resulted in a perceptual incongruency with visual and auditory stimuli, this allowed us to target perceptual variables of interest to our research question. Manikin models with the ability to vary tactile feedback could be considered to investigate the impact of tactile feedback on SMT delivery to older adults in future research.

## Conclusion

Using AI-generated case vignettes, this study found that contextual factors of aging had varying impacts on chiropractors’ kinetic and kinematic characteristics when delivering SMT. This suggests that clinicians differentiate between chronological, pathological, and felt age, and use that information to inform the kinetic and kinematic parameters of FBM administered to older adults. Future research is needed to identify ideal kinetic and kinematic characteristics to achieve desired clinical outcomes based on various aspects of aging, and train providers of SMT to utilize contextual factors to individualize care.

## Supplementary Information

Supplementary Material 1.

## Data Availability

Data is provided within the manuscript or supplementary information files. The datasets used and/or analysed during the current study are available from the corresponding author on reasonable request.

## Abbreviations

ANOVA: Analysis of variance
FBM: Force-based manipulation
FSTT®: Force sensing table technology
HAM®: Human analogue manikin
HVLA: High velocity low amplitude
IMU: Inertial motion units
NWHSU: Northwestern Health Sciences University
SMT: Spinal manipulative therapy
