# Supplementary material for: Contextual factors related to aging determine force-based manipulation dosage: a prospective cross-sectional study
Source: Chiropr Man Therap. 2025 May 21;33:20. doi: 10.1186/s12998-025-00584-1 (PMC12093877; doi:10.1186/s12998-025-00584-1)
Supplement: Supplementary file 1 — Supplementary Material 1. [file 12998_2025_584_MOESM1_ESM.pdf]

This 35-year-old patient presents with uncomplicated, 5/10 mid thoracic pain, 3 months in duration.

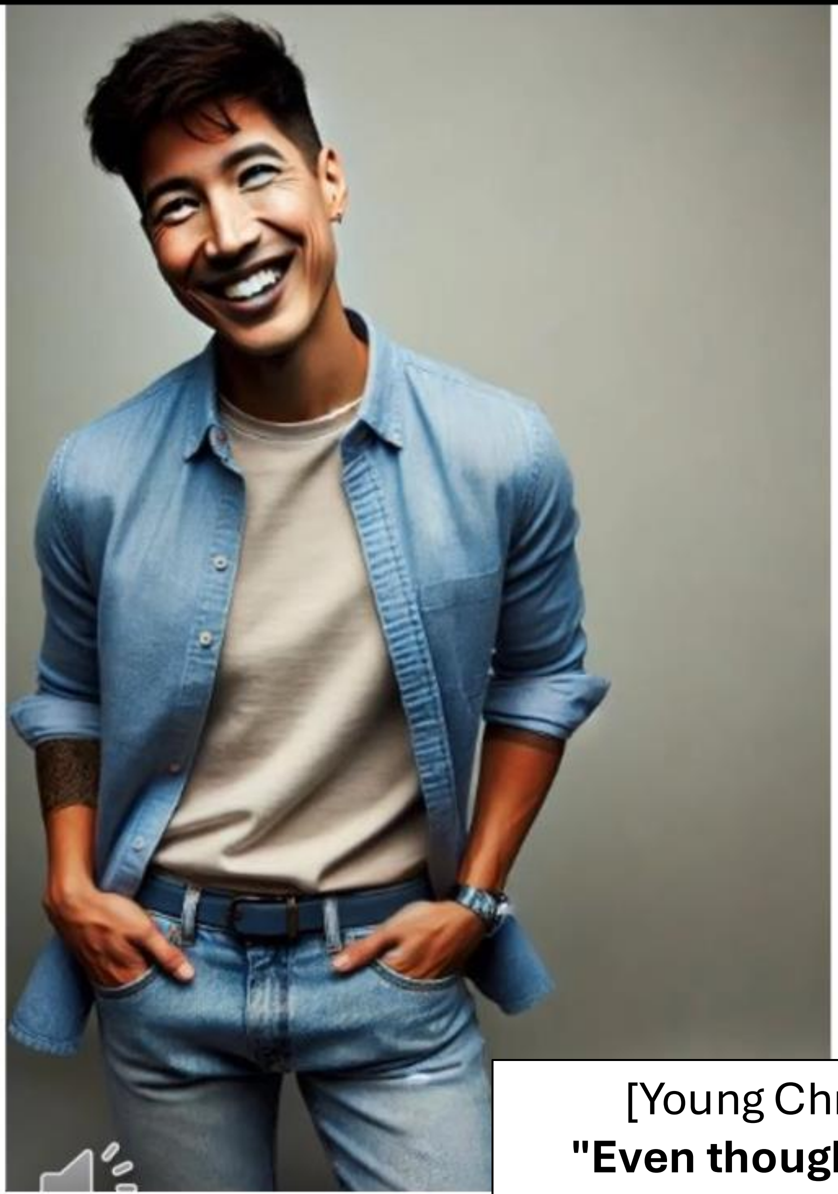

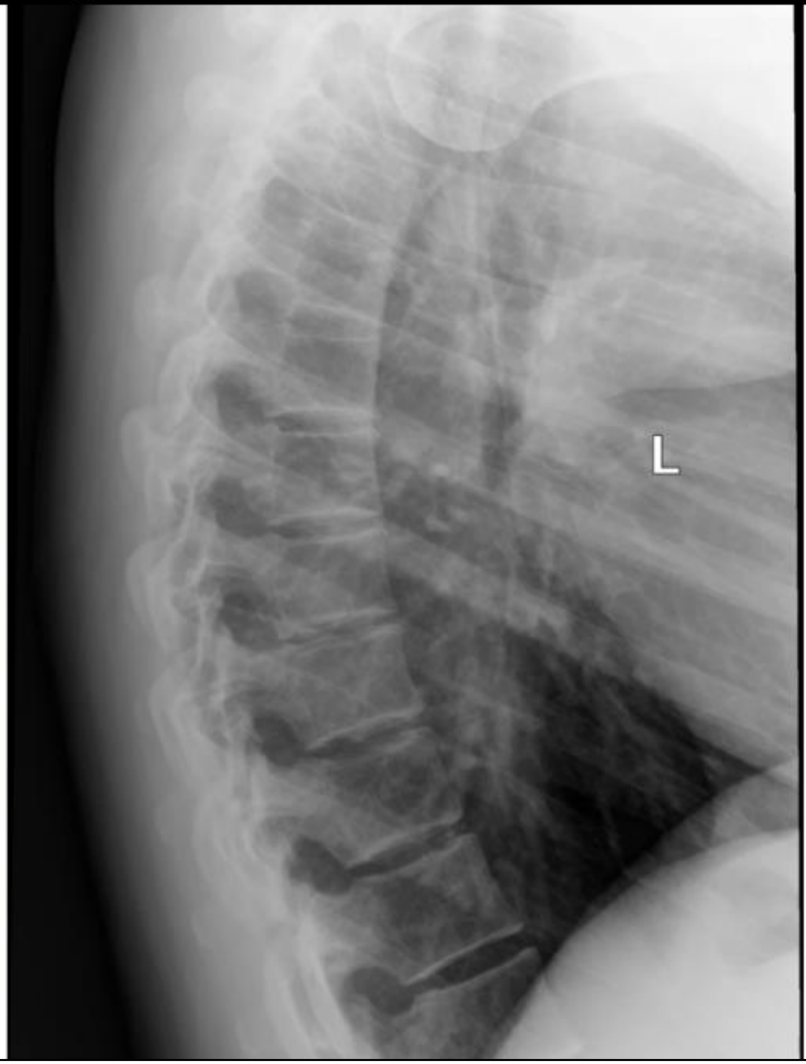

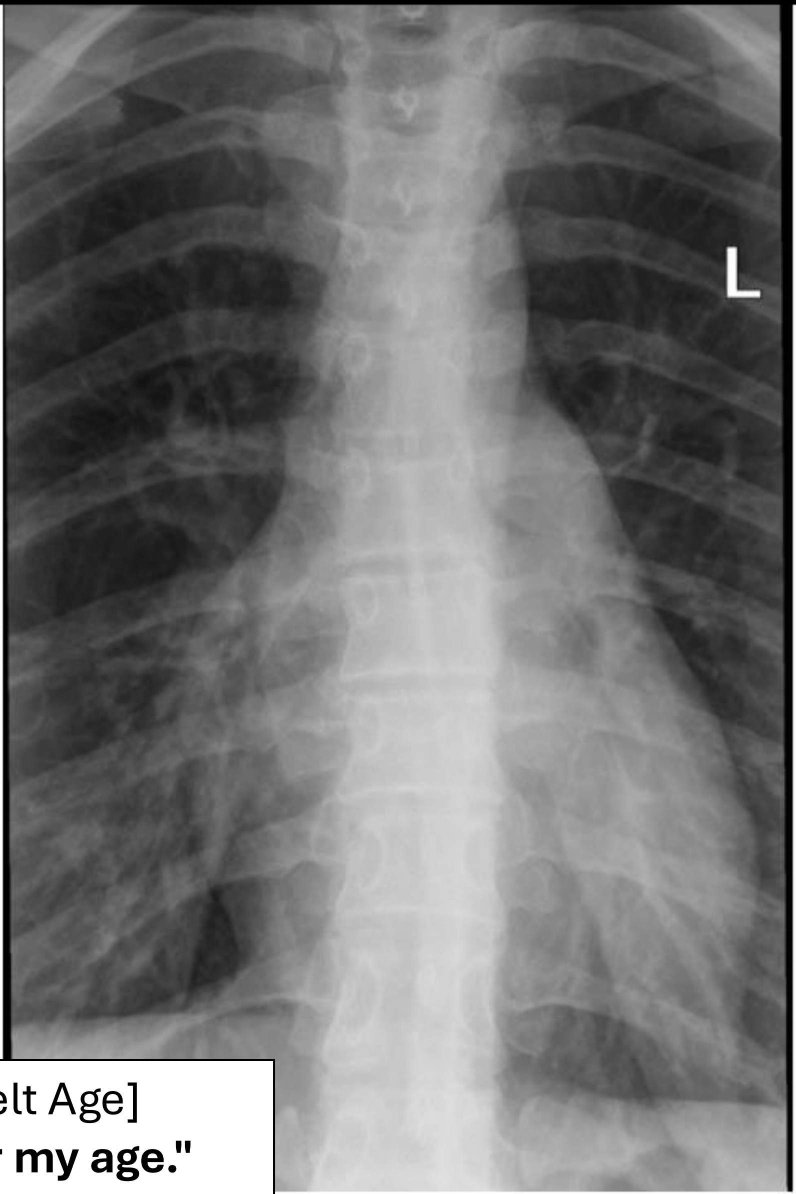

[Young Chronological/Young Pathological/Young Felt Age]

"Even though I have back pain, I feel really good for my age."

This 35-year-old patient presents with uncomplicated, 5/10 mid thoracic pain, 3 months in duration.

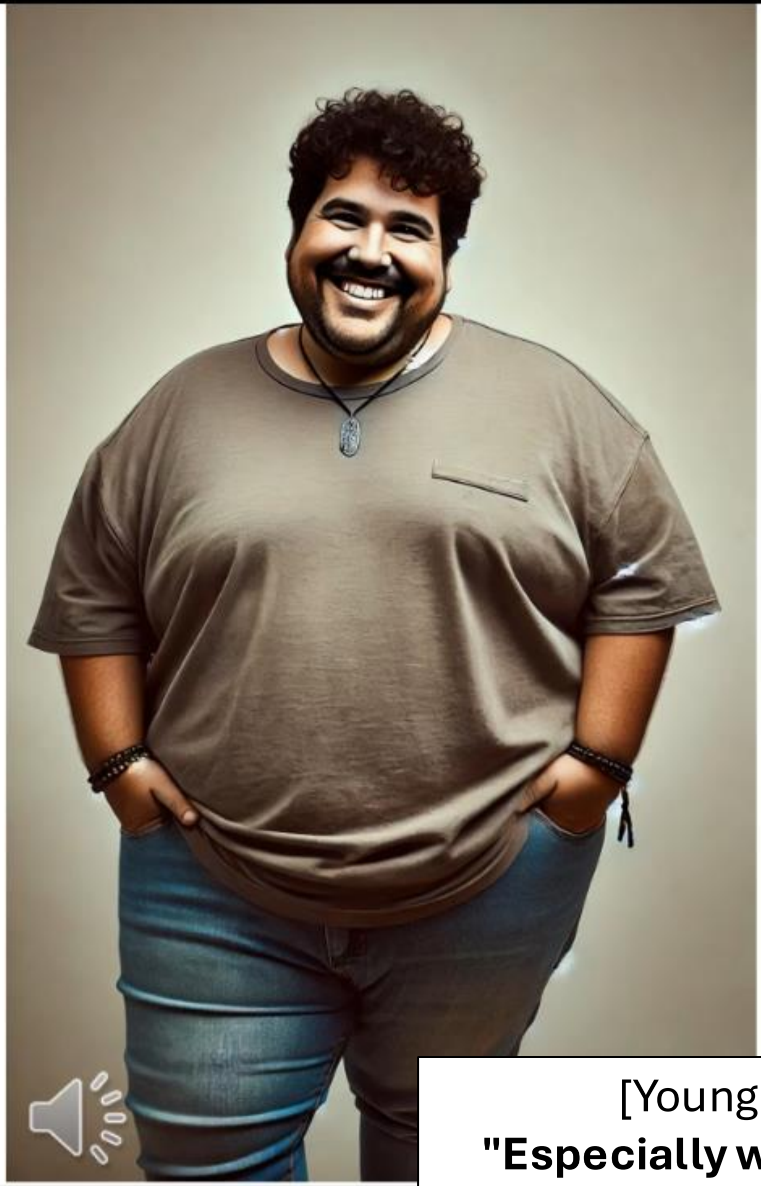

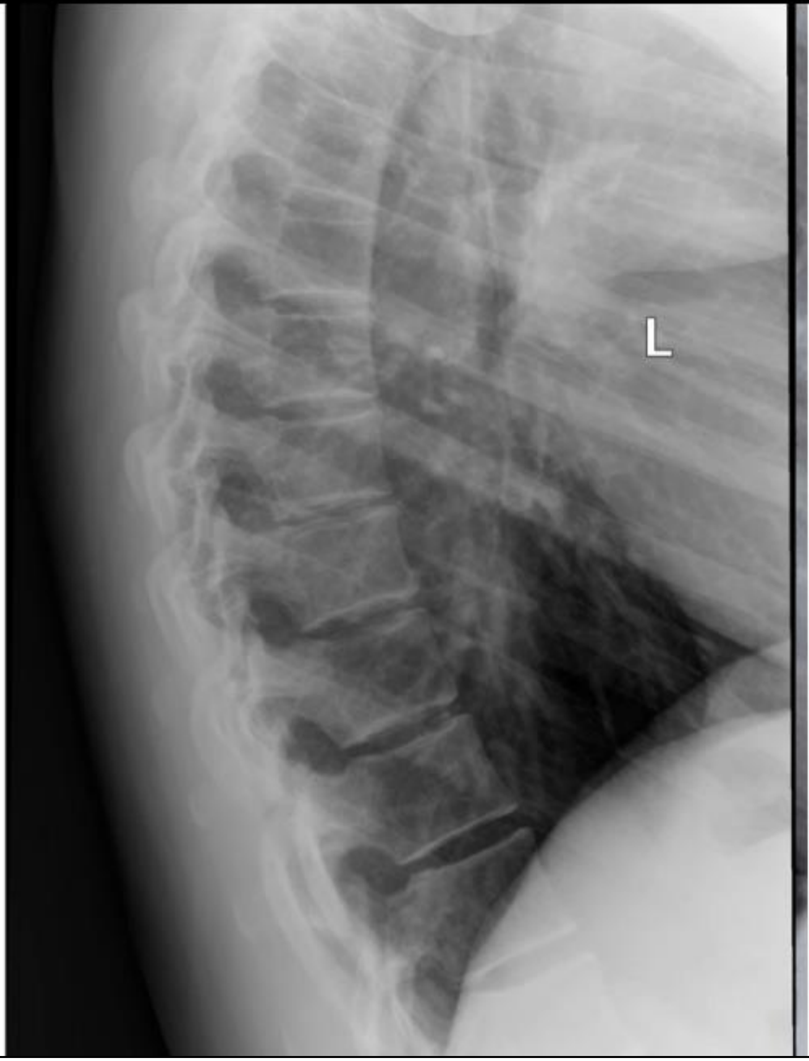

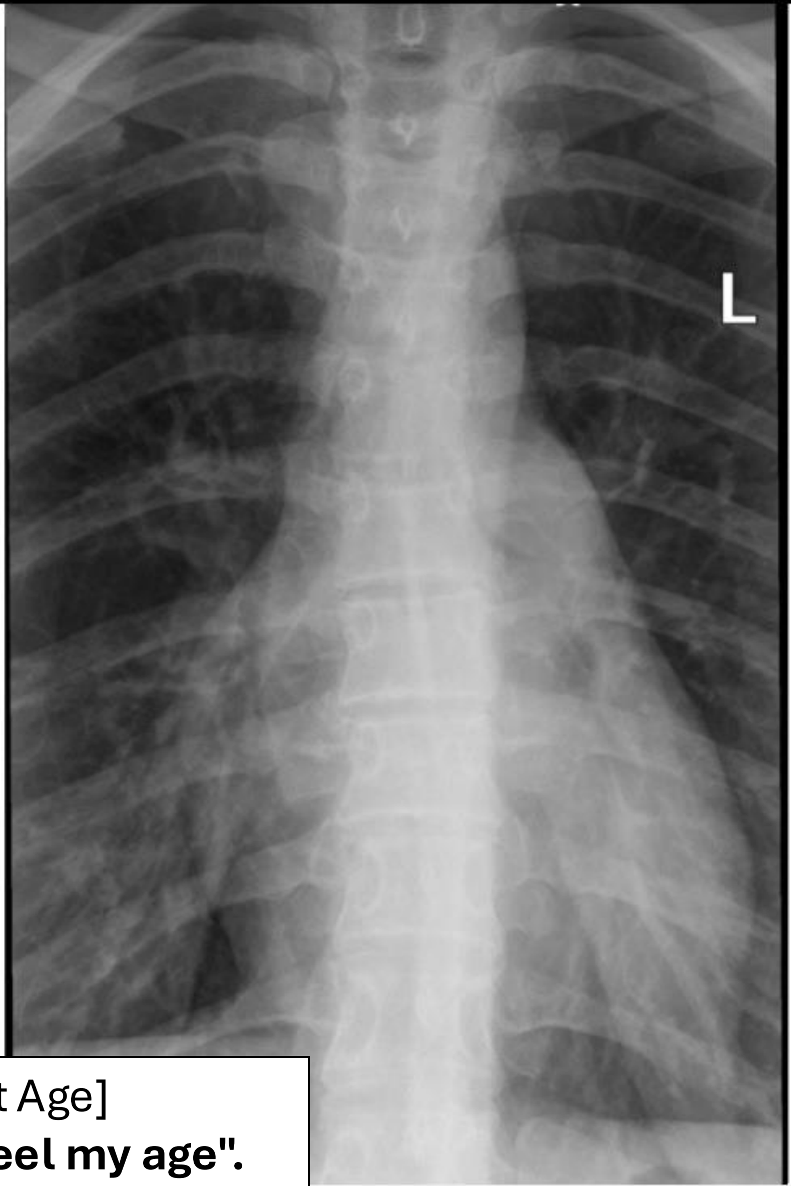

[Young Chronological/Young Pathological/Old Felt Age]
"Especially with this back pain, I'm really starting to feel my age".

This 35-year-old patient presents with uncomplicated, 5/10 mid thoracic pain, 3 months in duration.

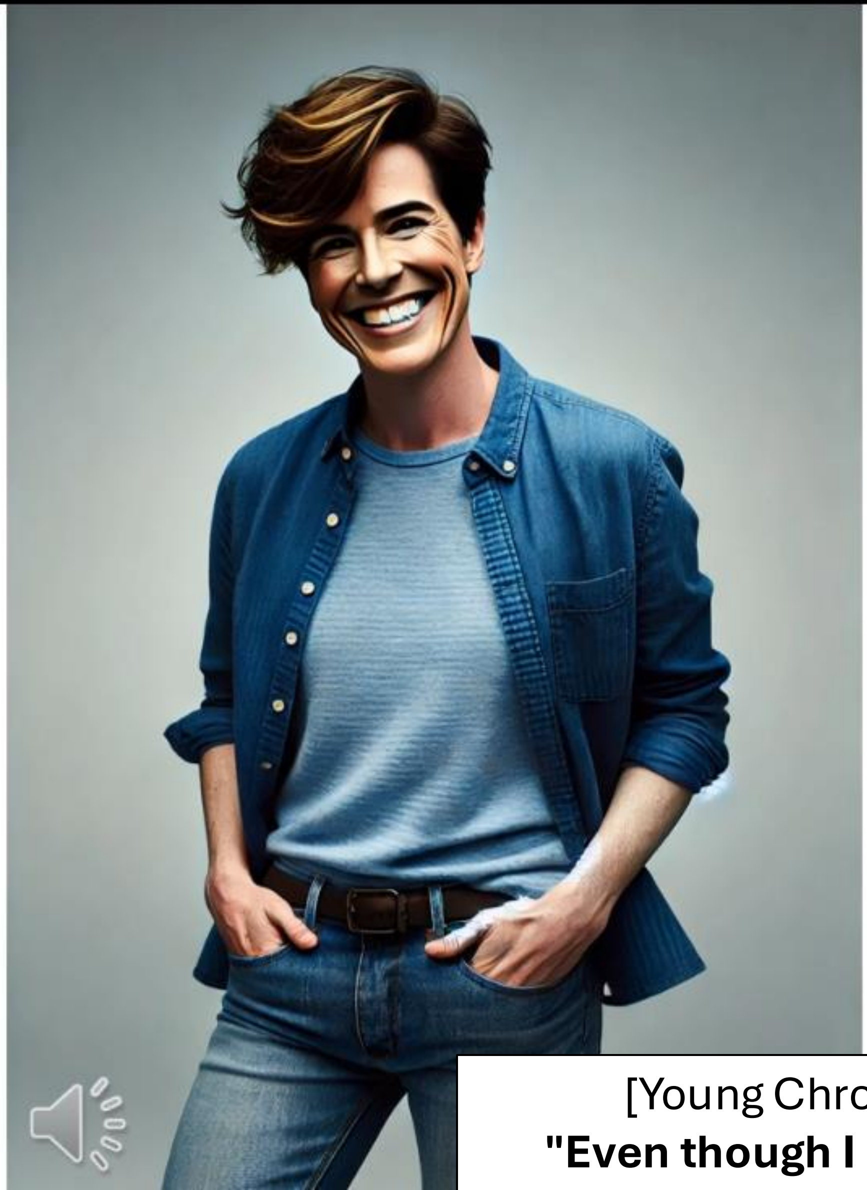

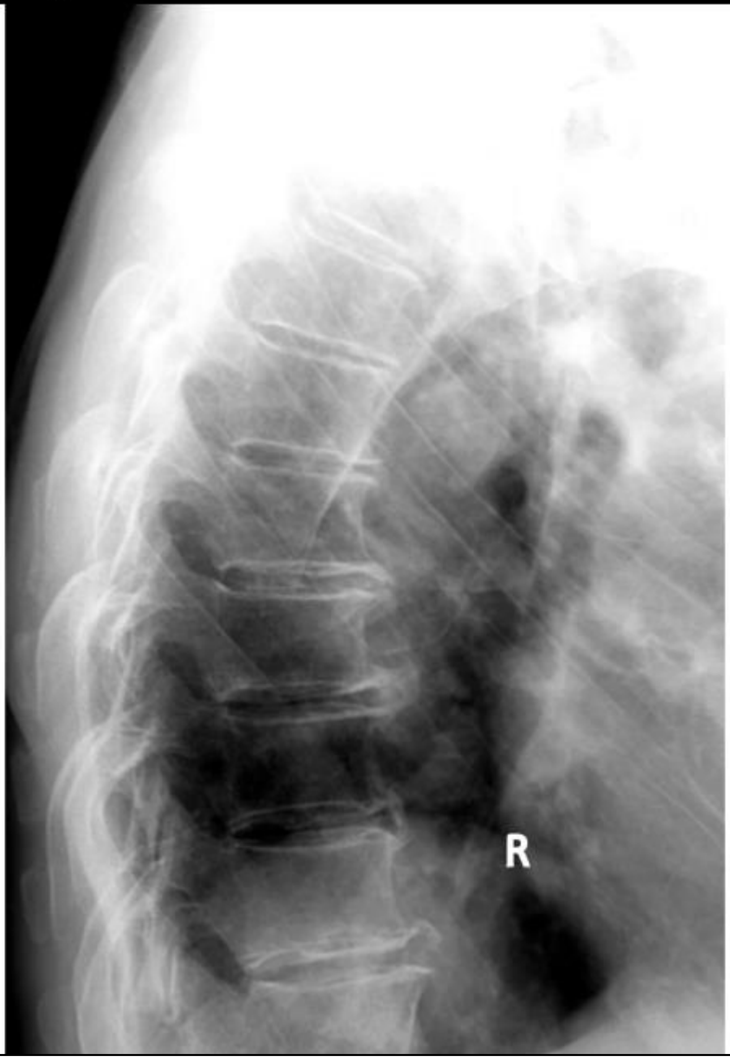

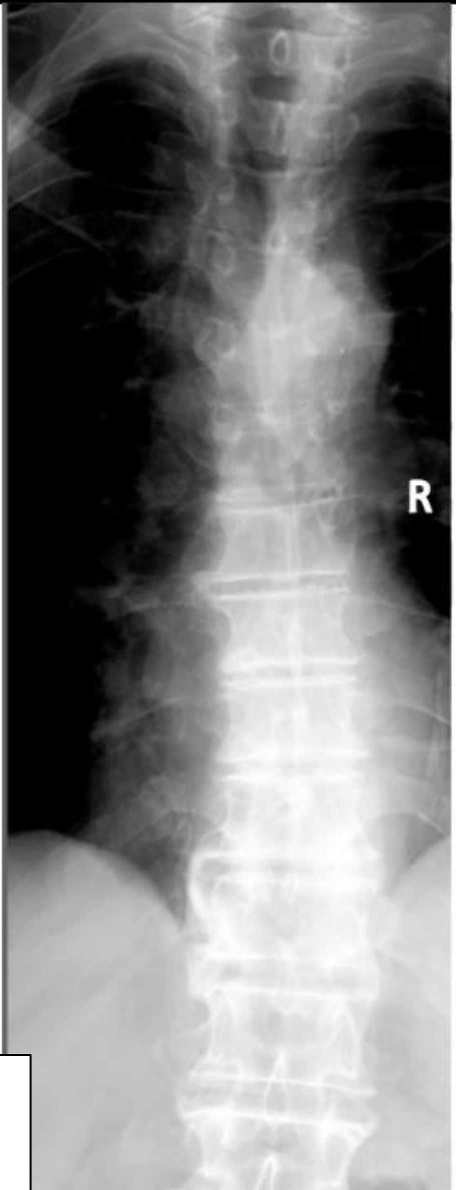

[Young Chronological/Old Pathological/Young Felt Age]

"Even though I have back pain, I feel really good for my age."

This 35-year-old patient presents with uncomplicated, 5/10 mid thoracic pain, 3 months in duration.

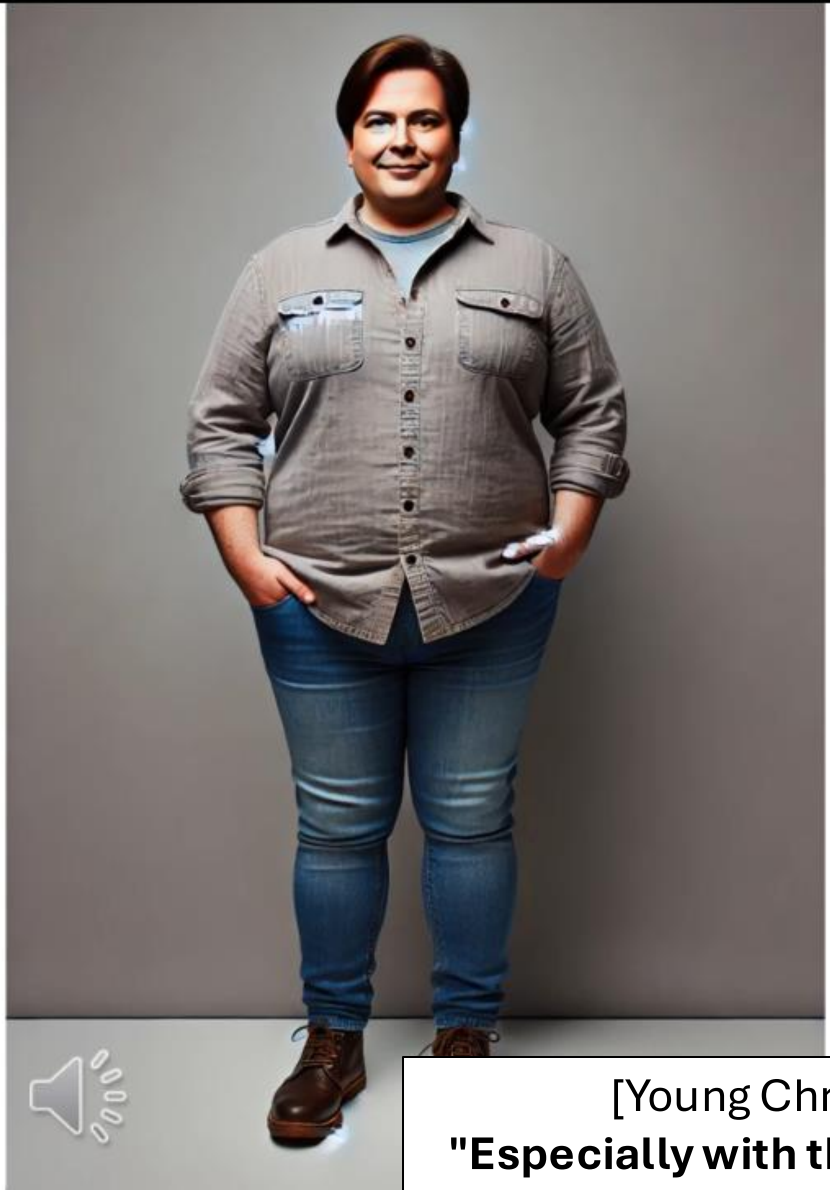

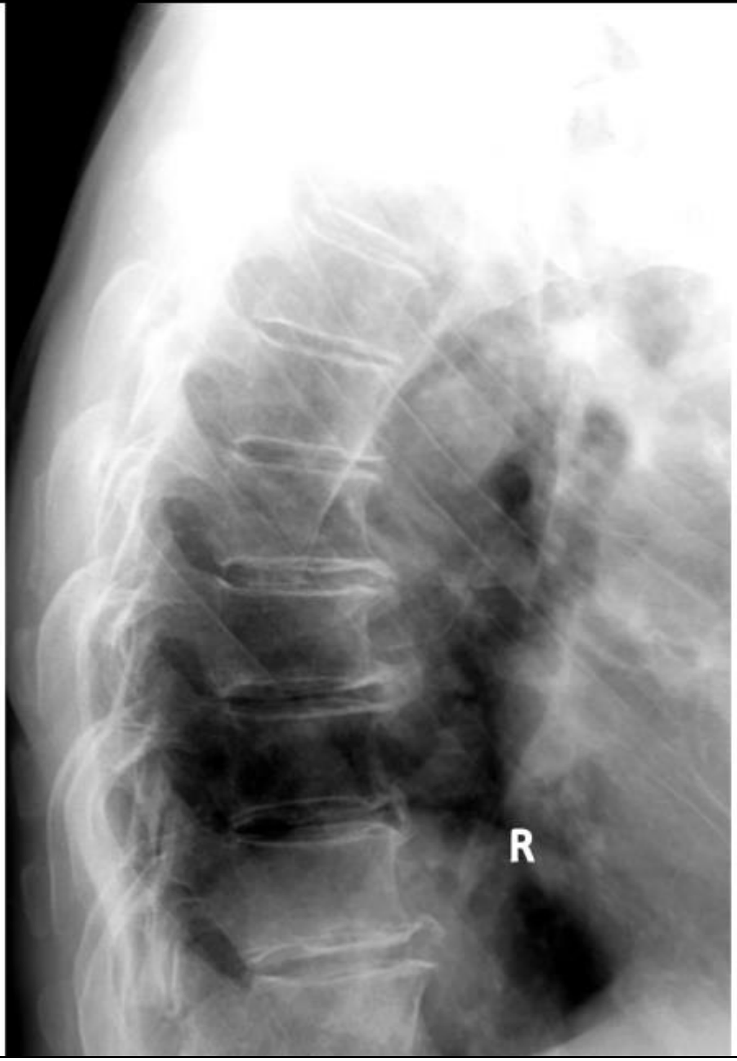

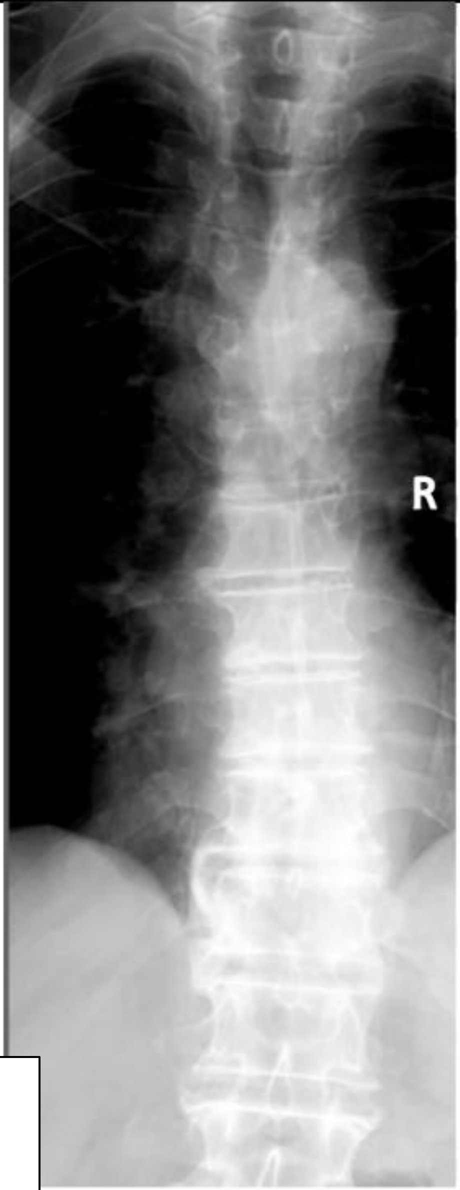

[Young Chronological/Old Pathological/Old Felt Age]
"Especially with this back pain, I'm really starting to feel my age".

This 65-year-old patient presents with uncomplicated, 5/10 mid thoracic pain, 3 months in duration.

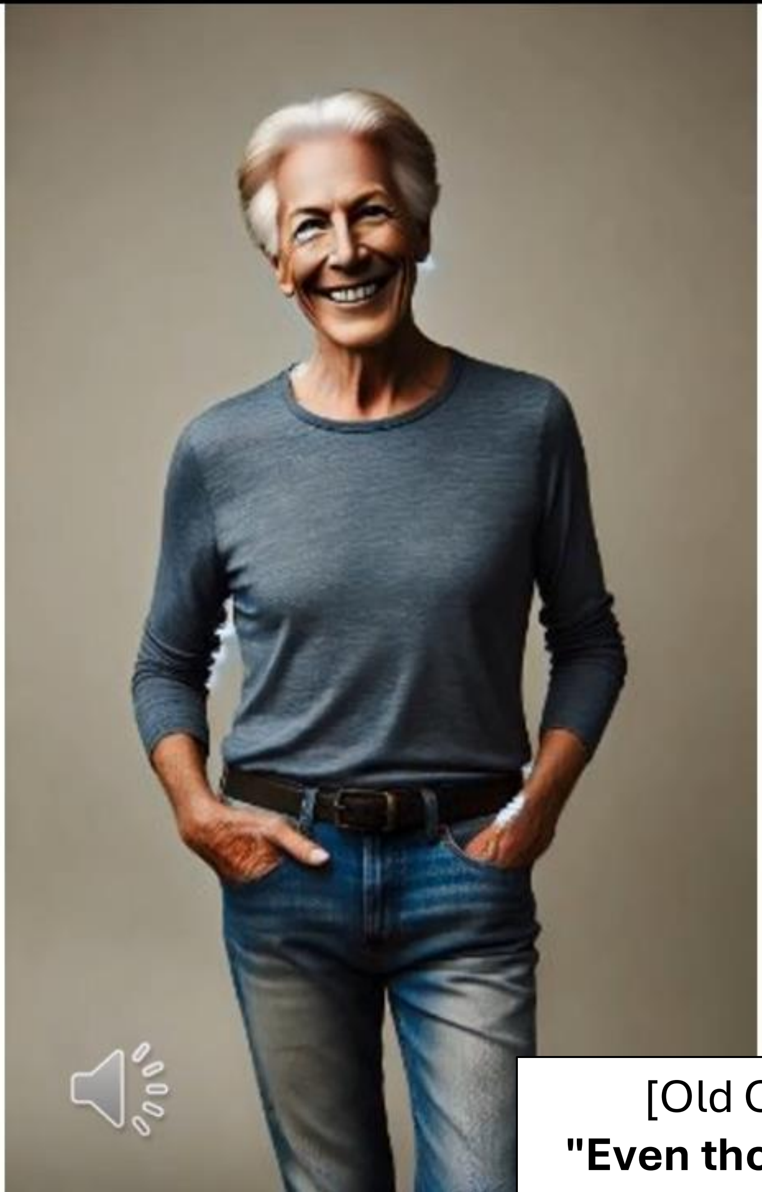

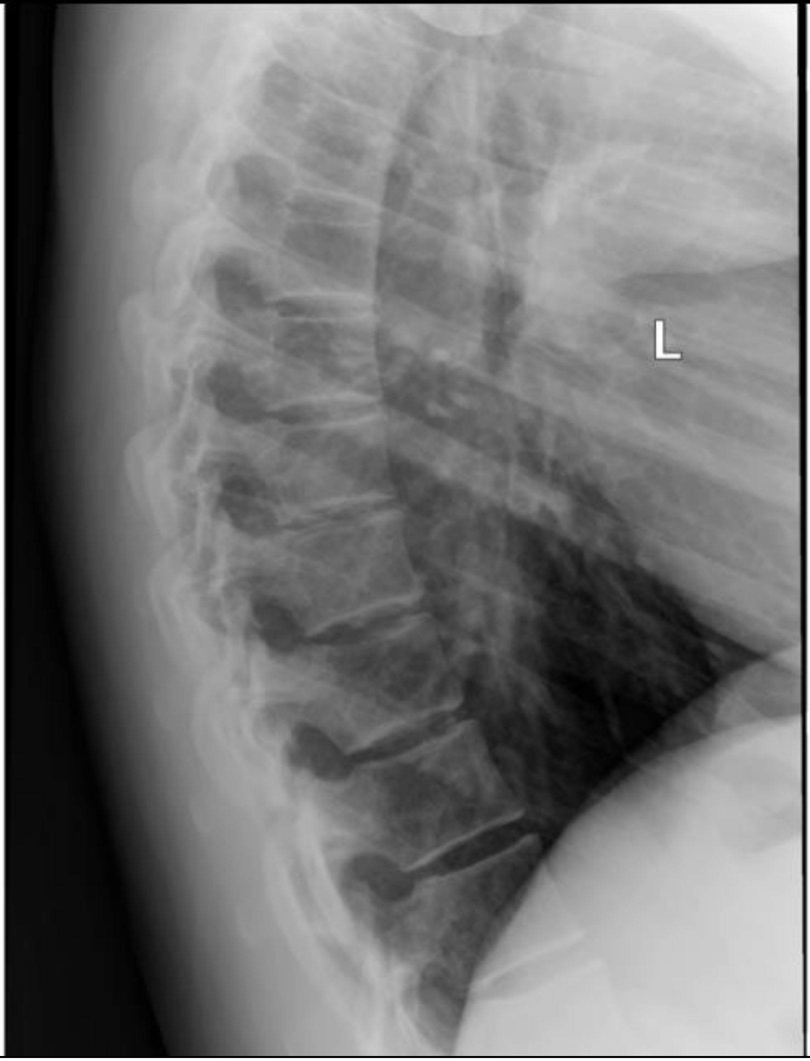

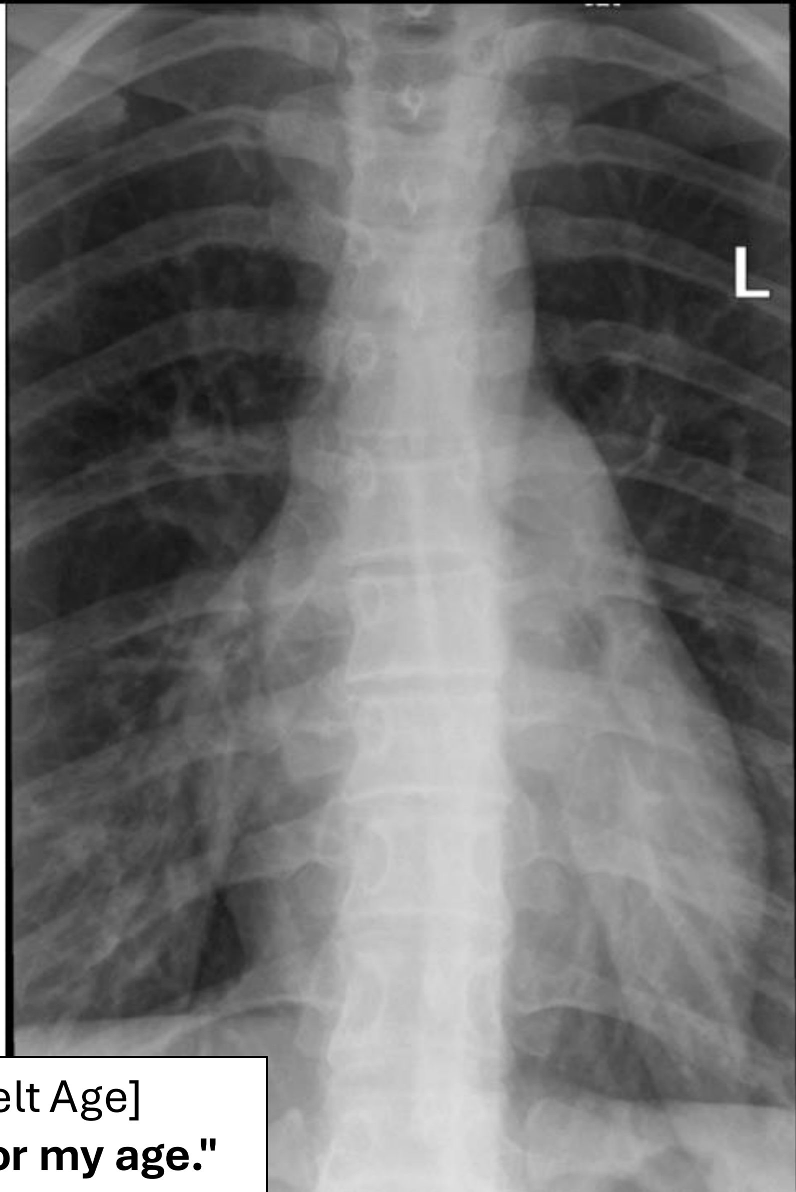

[Old Chronological/Young Pathological/Young Felt Age]

"Even though I have back pain, I feel really good for my age."

This 65-year-old patient presents with uncomplicated, 5/10 mid thoracic pain, 3 months in duration.

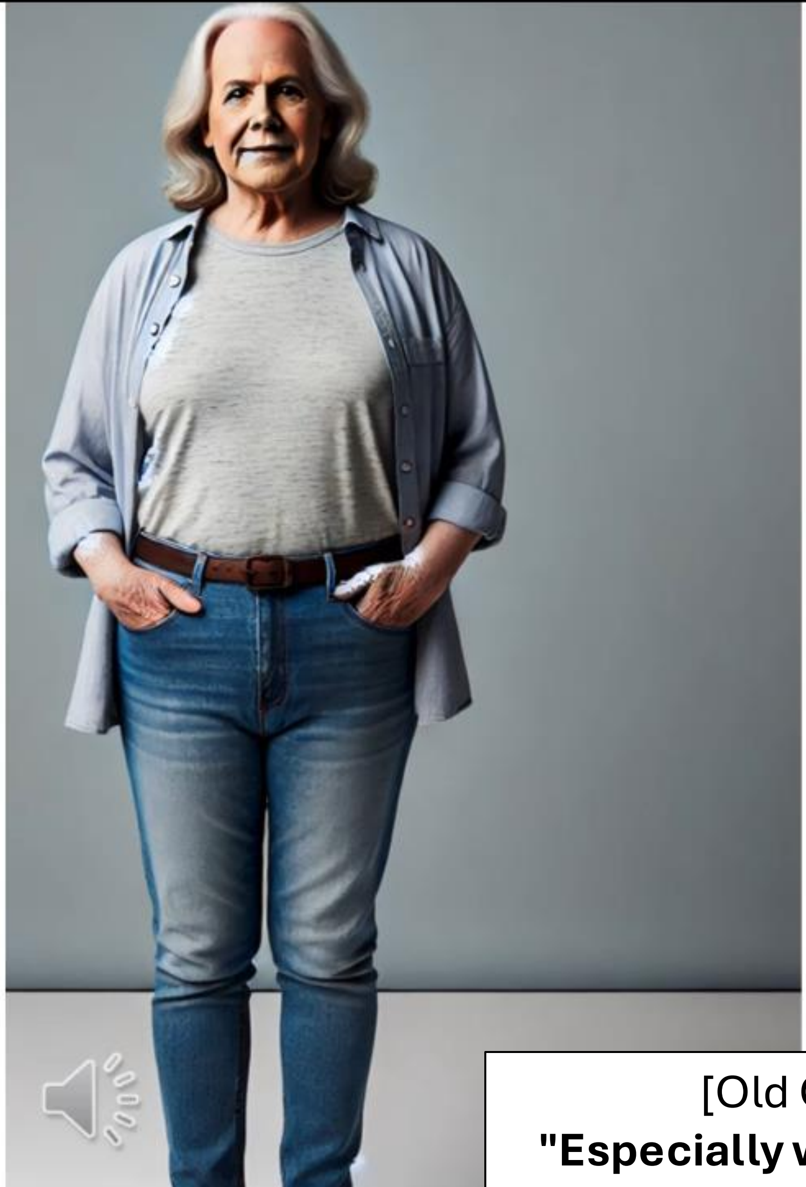

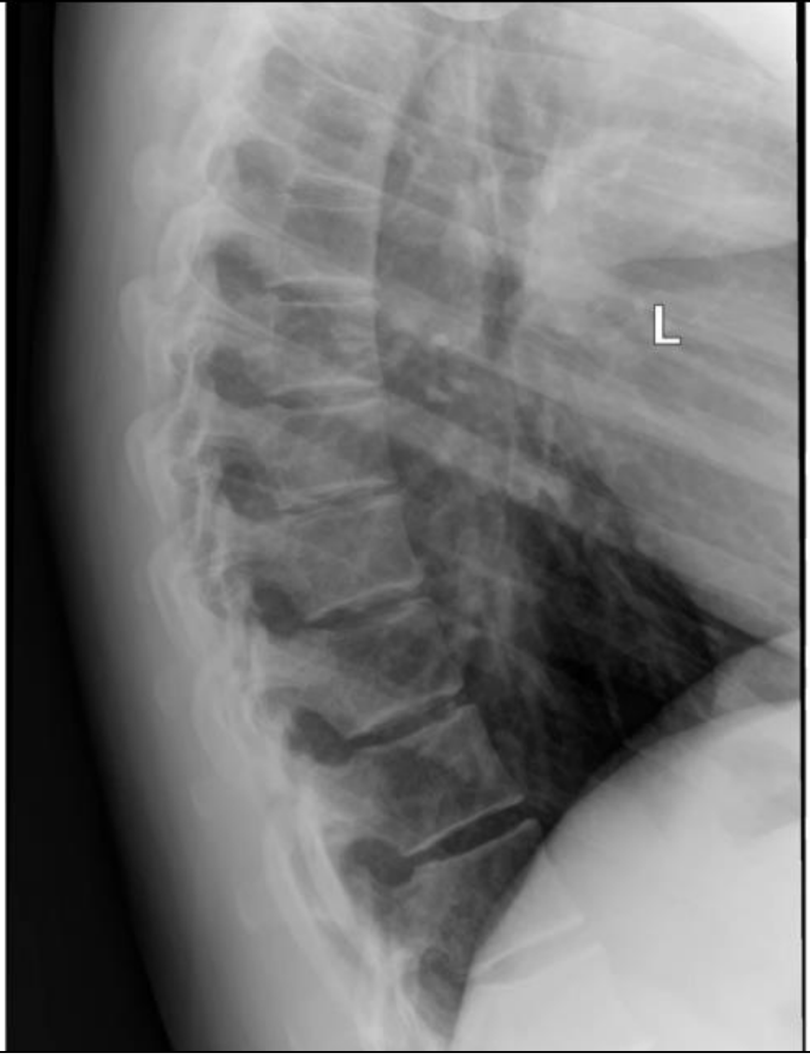

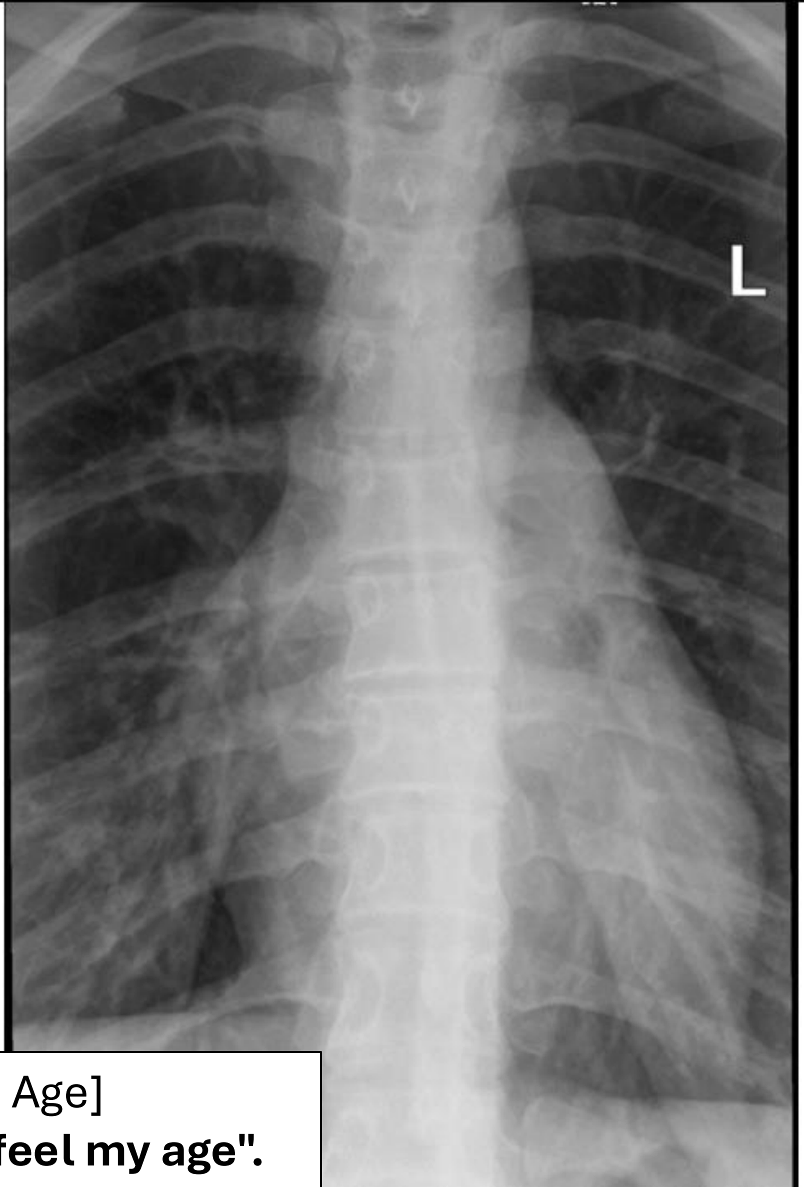

[Old Chronological/Young Pathological/Old Felt Age]

"Especially with this back pain, I'm really starting to feel my age".

This 65-year-old patient presents with uncomplicated, 5/10 mid thoracic pain, 3 months in duration.

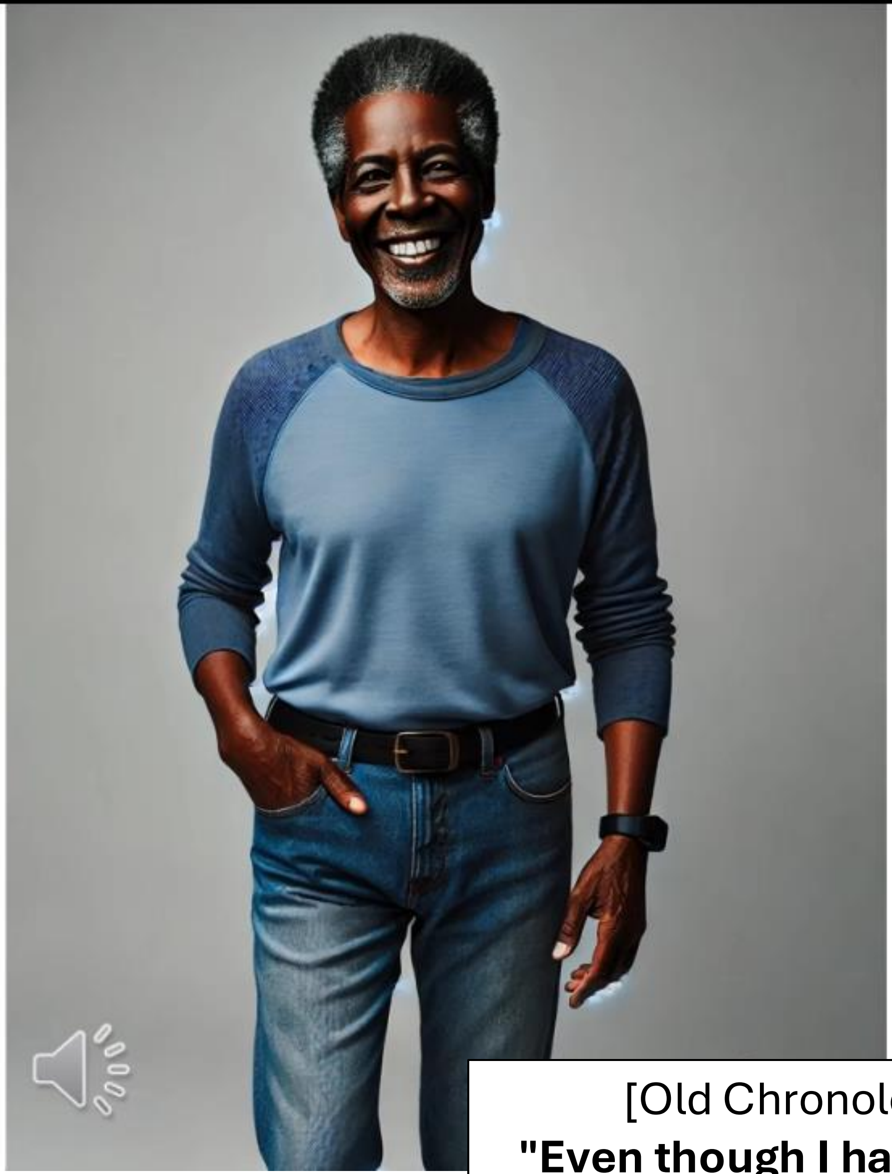

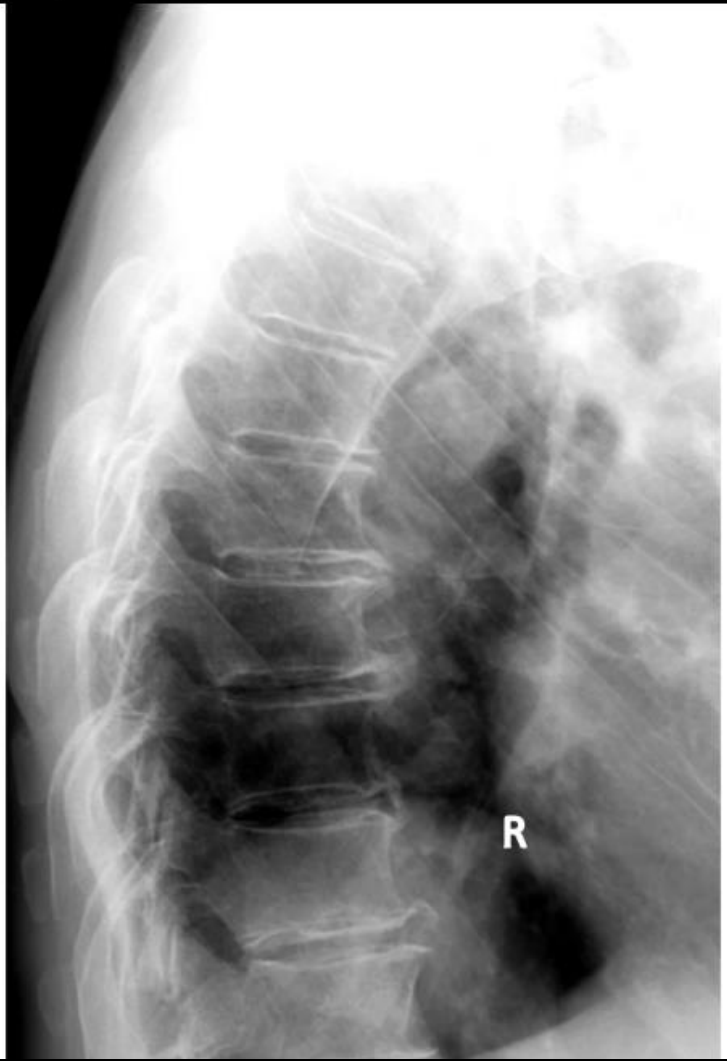

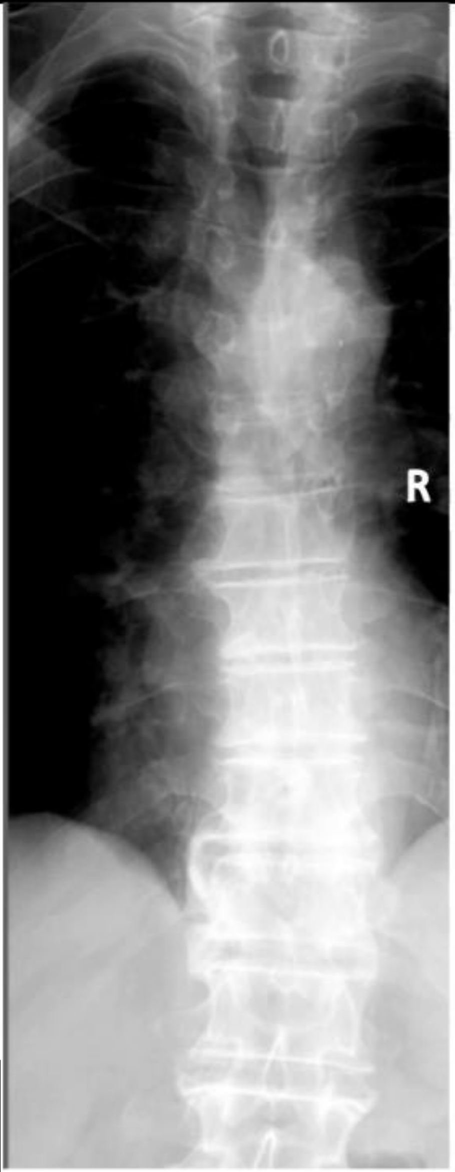

[Old Chronological/Old Pathological/Young Felt Age]

"Even though I have back pain, I feel really good for my age."

This 65-year-old patient presents with uncomplicated, 5/10 mid thoracic pain, 3 months in duration.

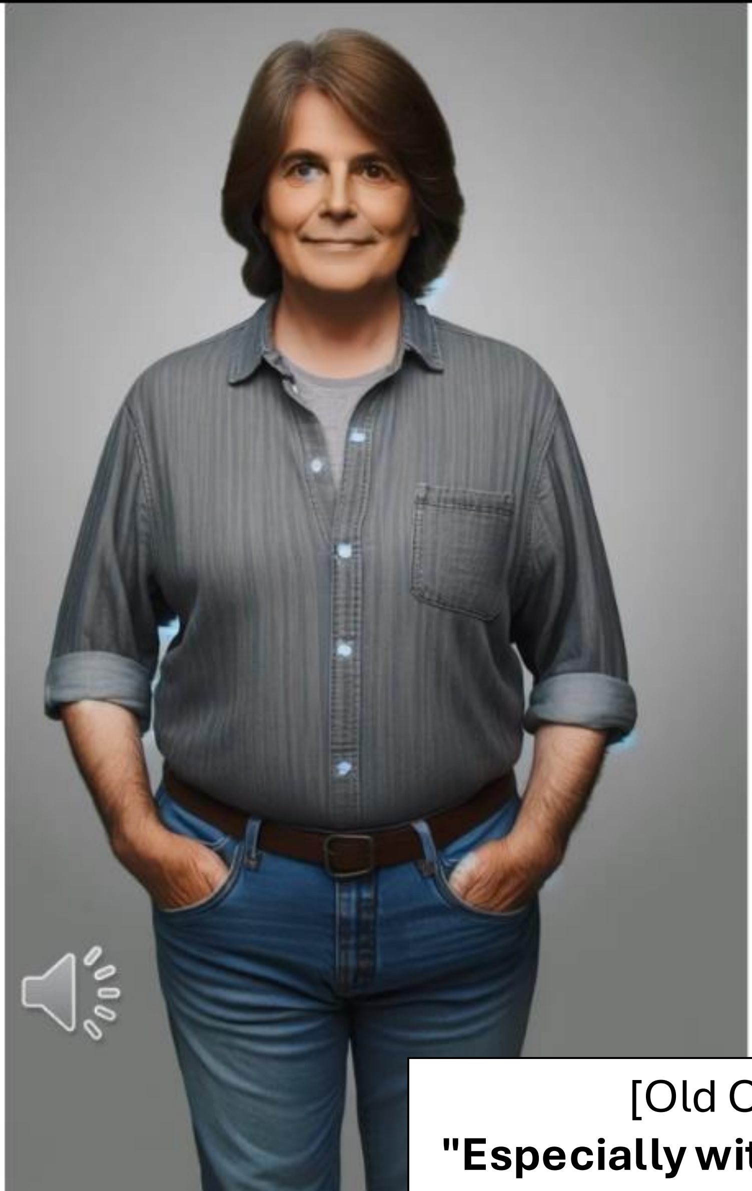

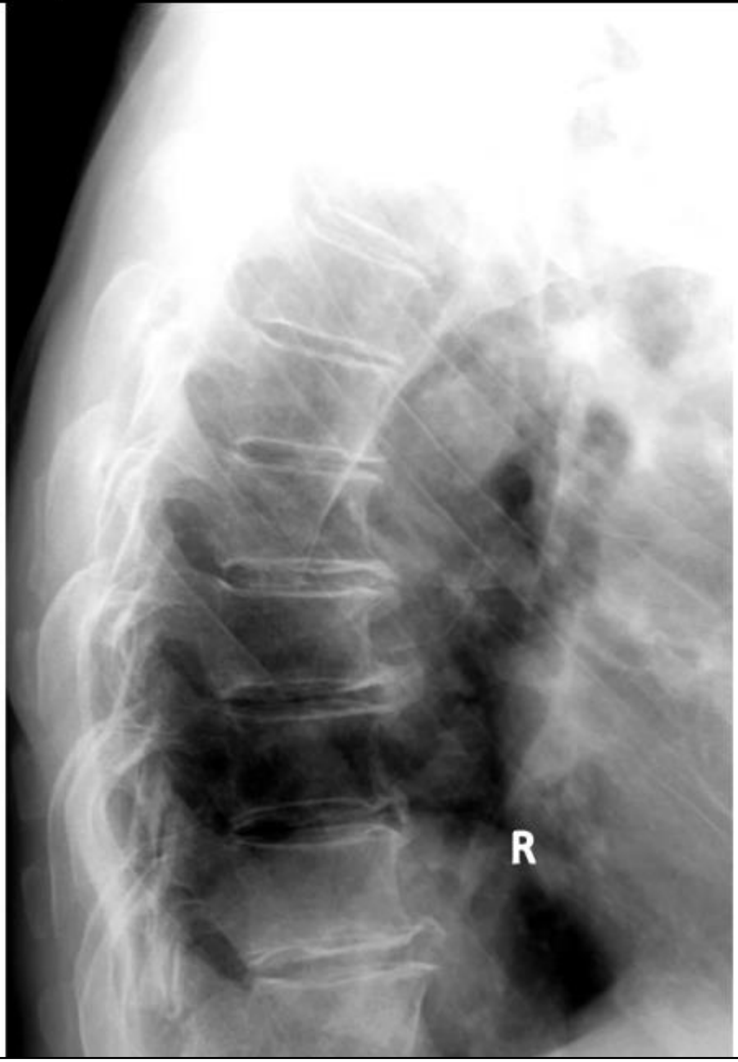

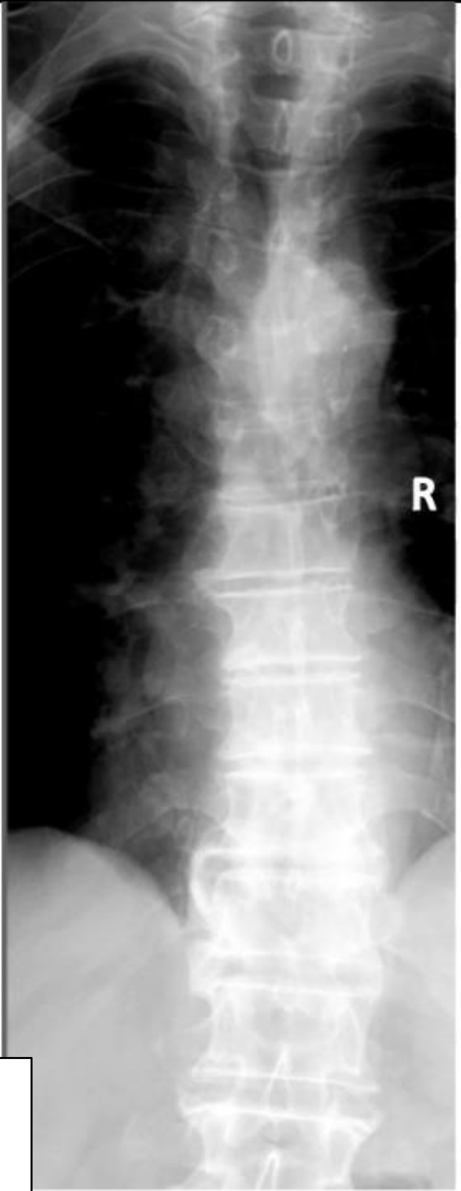

00:02

[Old Chronological/Old Pathological/Old Felt Age]

"Especially with this back pain, I'm really starting to feel my age".

This 85-year-old patient presents with uncomplicated, 5/10 mid thoracic pain, 3 months in duration.

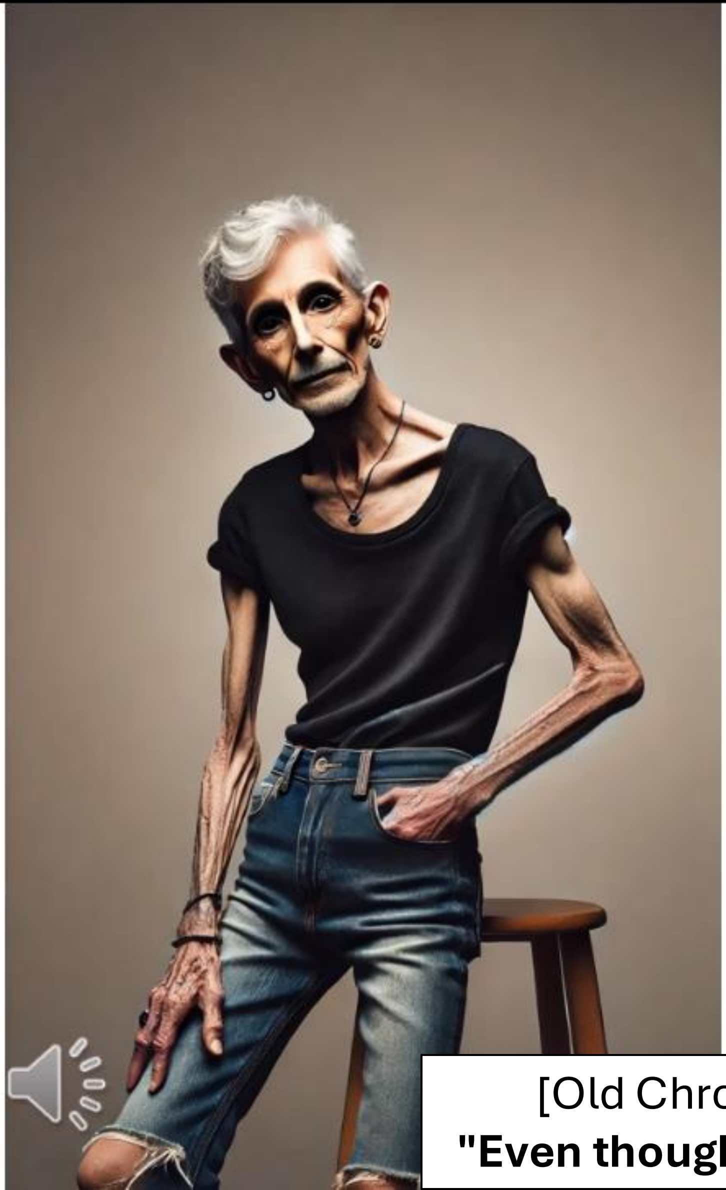

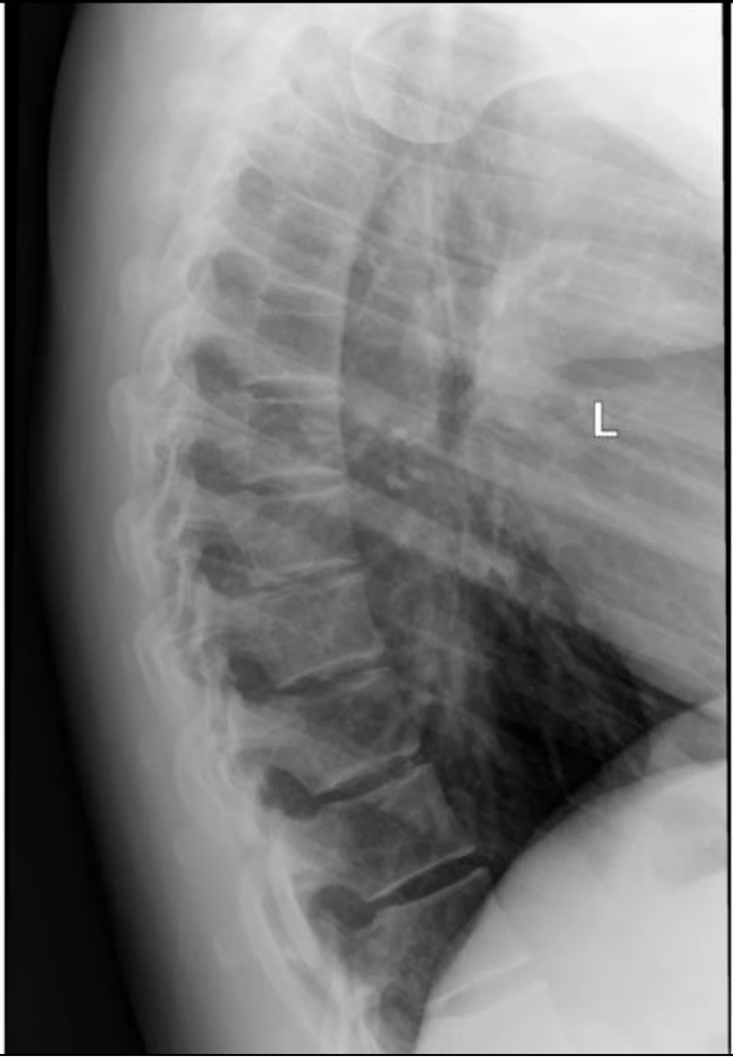

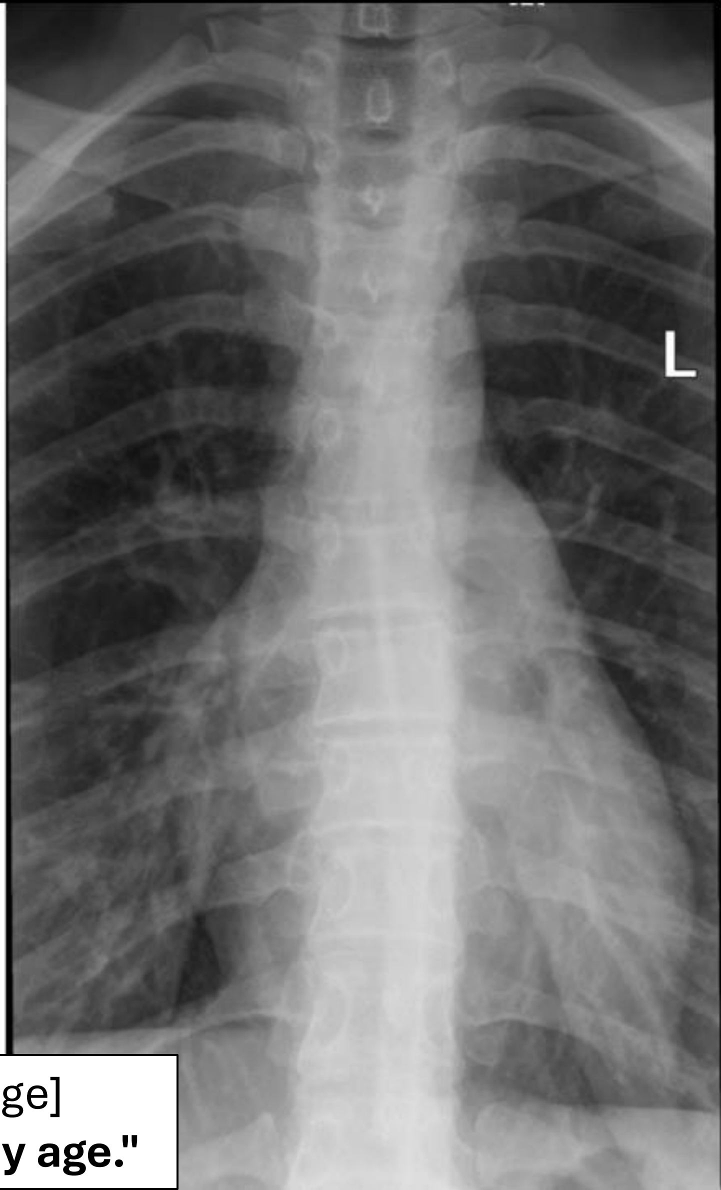

[Old Chronological/Young Pathological/Young Felt Age]
"Even though I have back pain, I feel really good for my age."

This 85-year-old patient presents with uncomplicated, 5/10 mid thoracic pain, 3 months in duration.

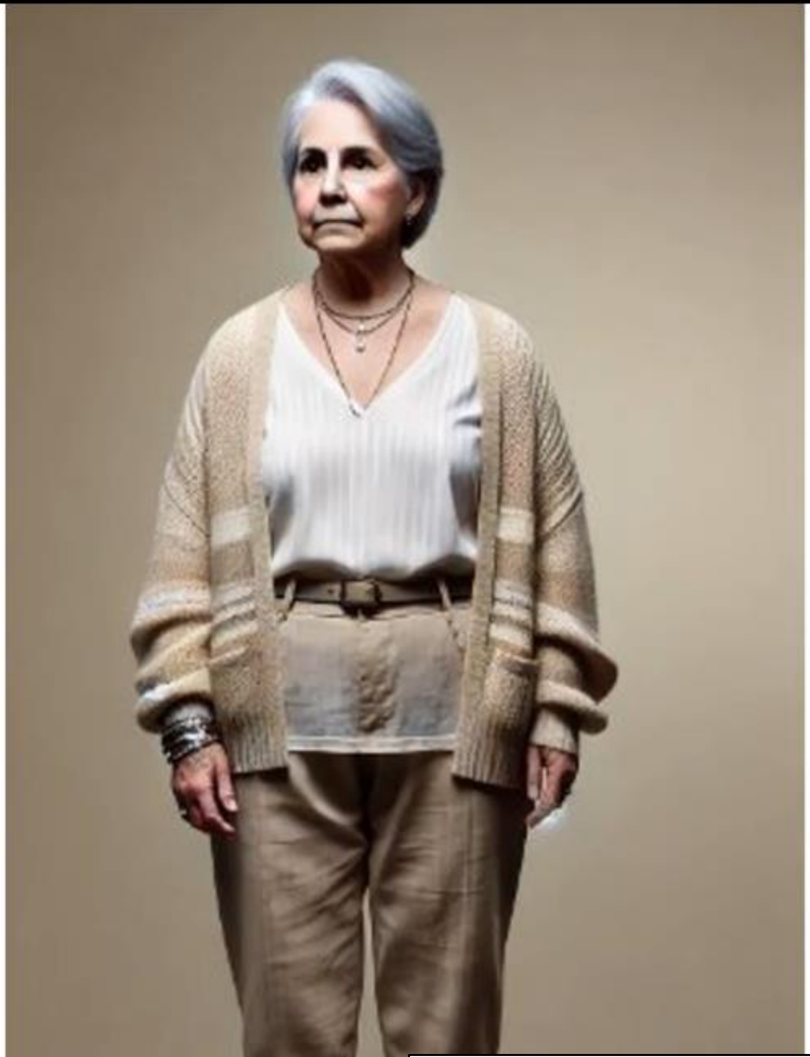

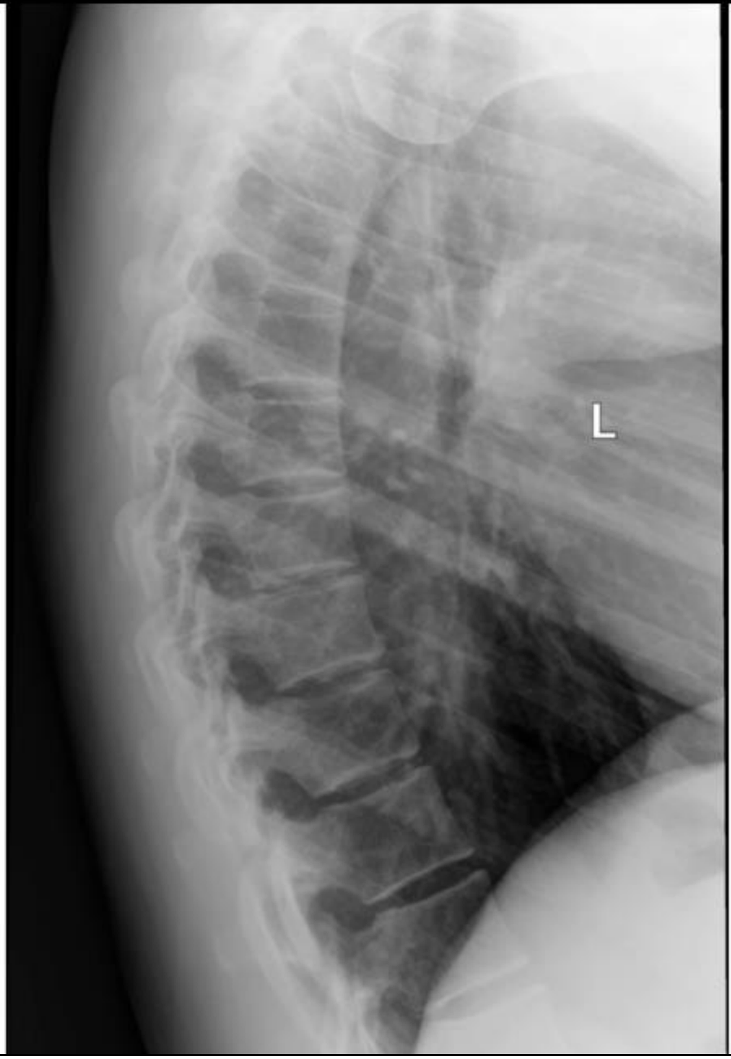

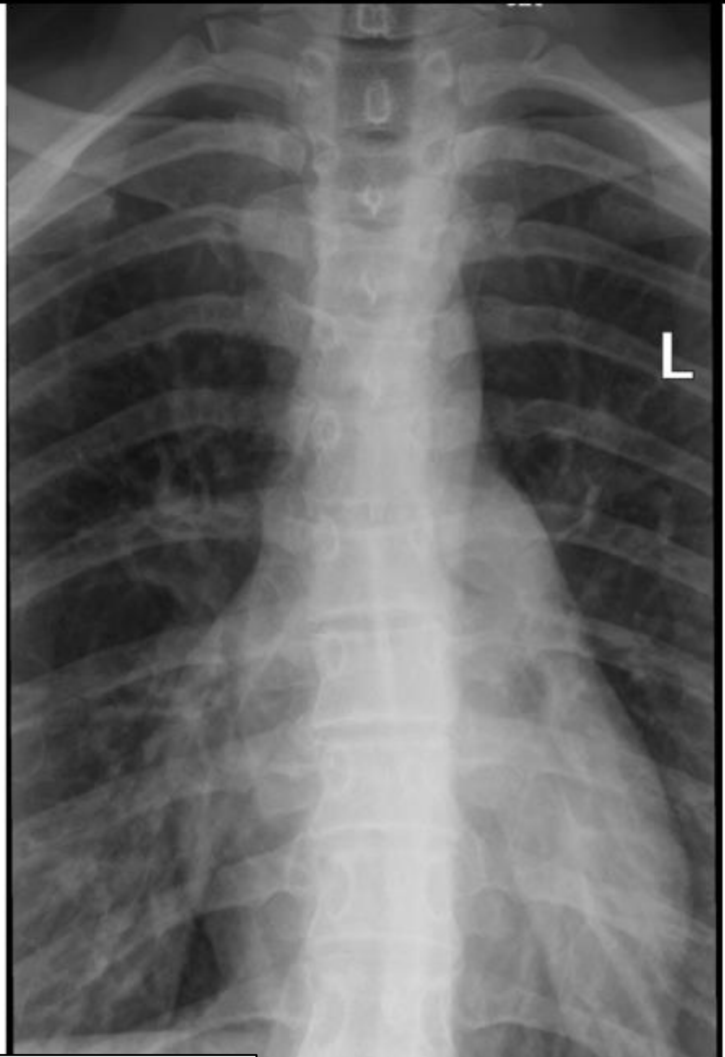

[Old Chronological/Young Pathological/Old Felt Age]"

"Especially with this back pain, I'm really starting to feel my age."

This 85-year-old patient presents with uncomplicated, 5/10 mid thoracic pain, 3 months in duration.

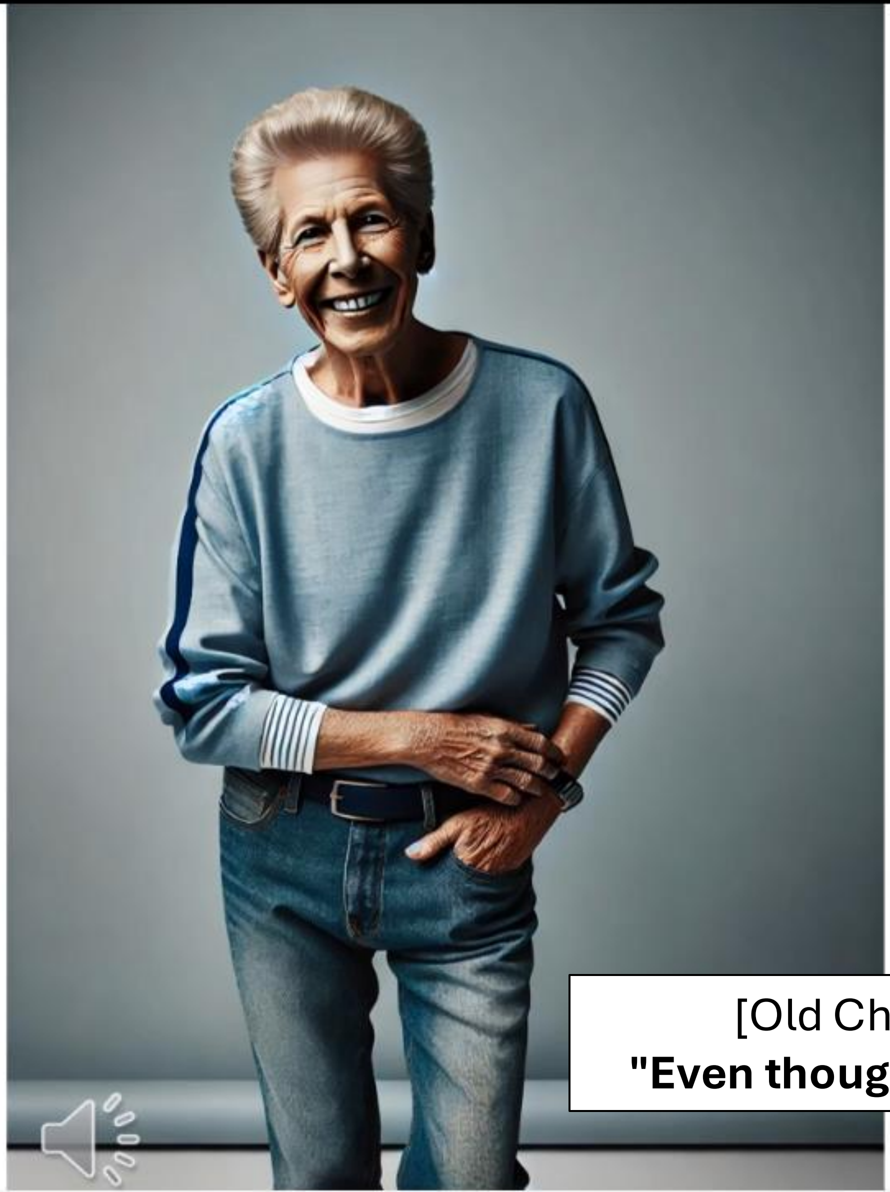

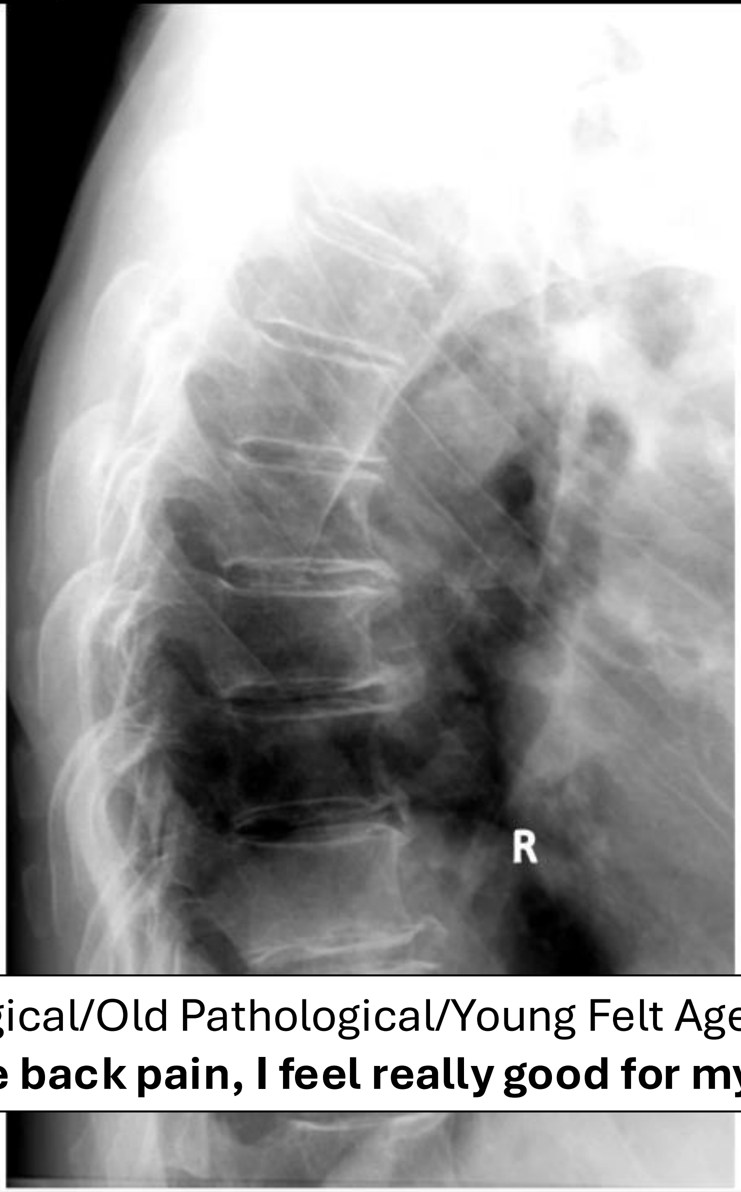

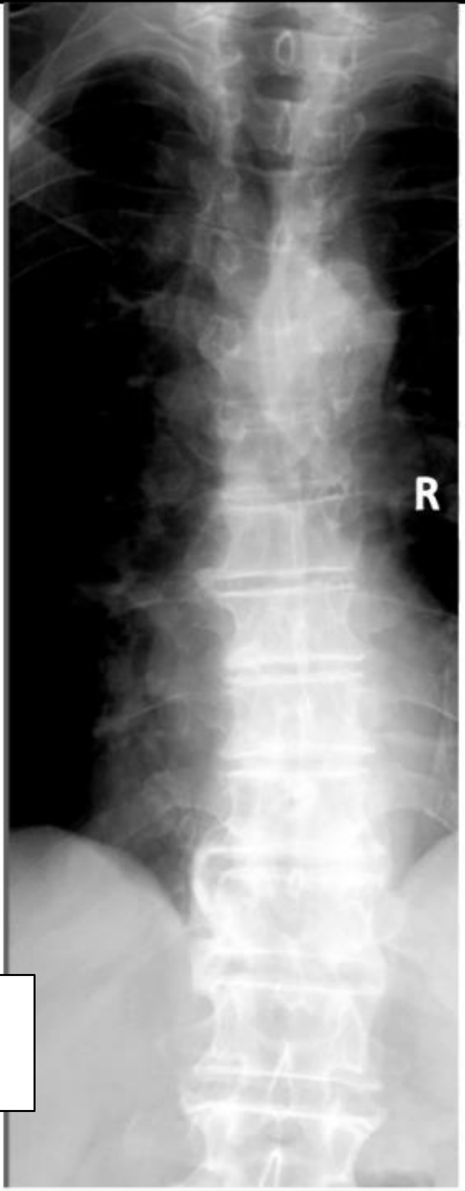

[Old Chronological/Old Pathological/Young Felt Age]

"Even though I have back pain, I feel really good for my age."

This 85-year-old patient presents with uncomplicated, 5/10 mid thoracic pain, 3 months in duration.

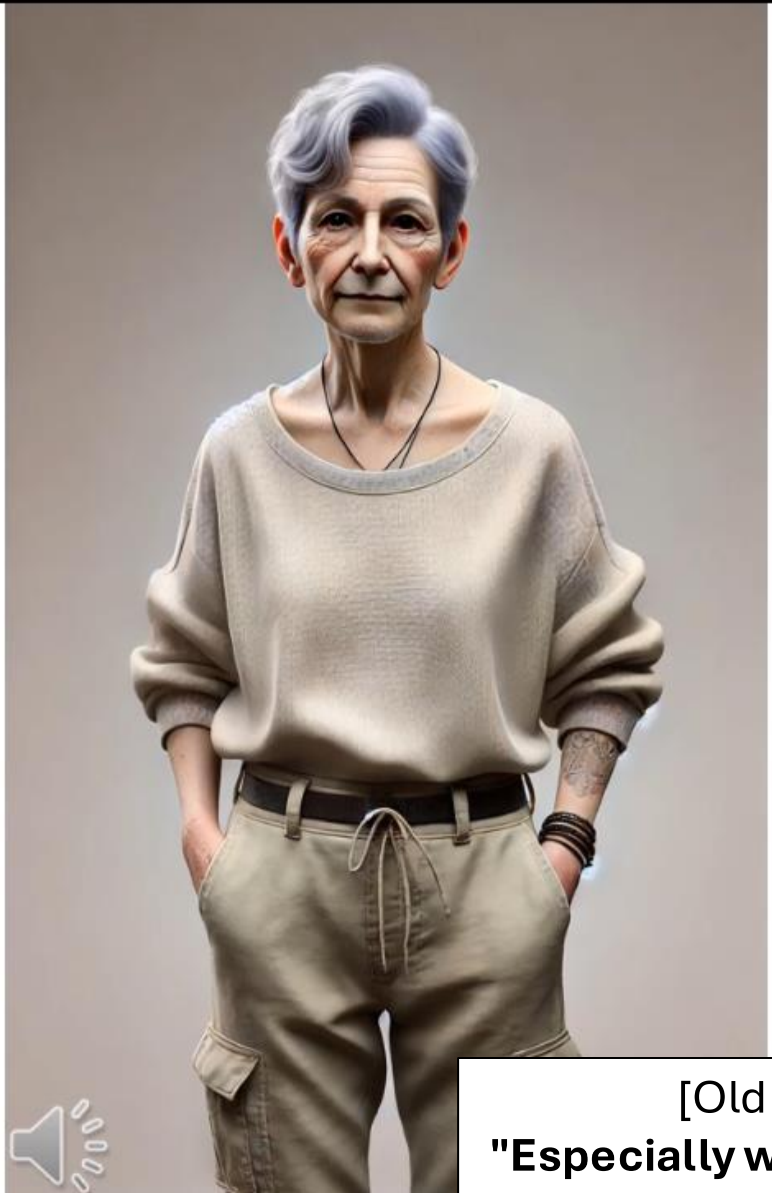

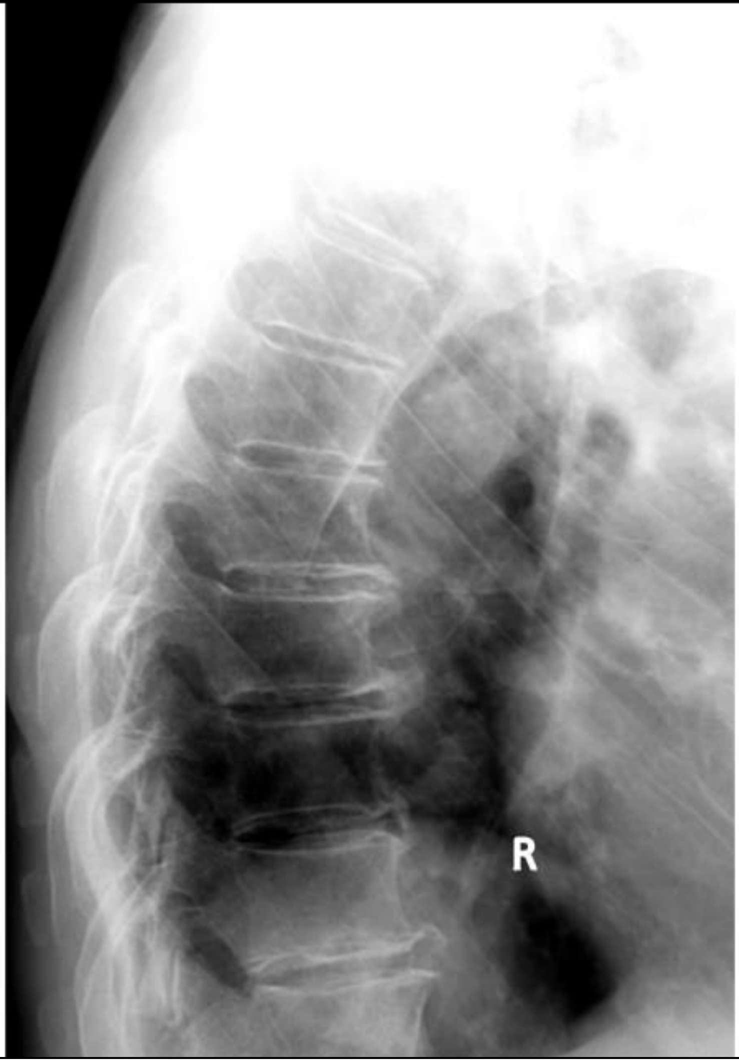

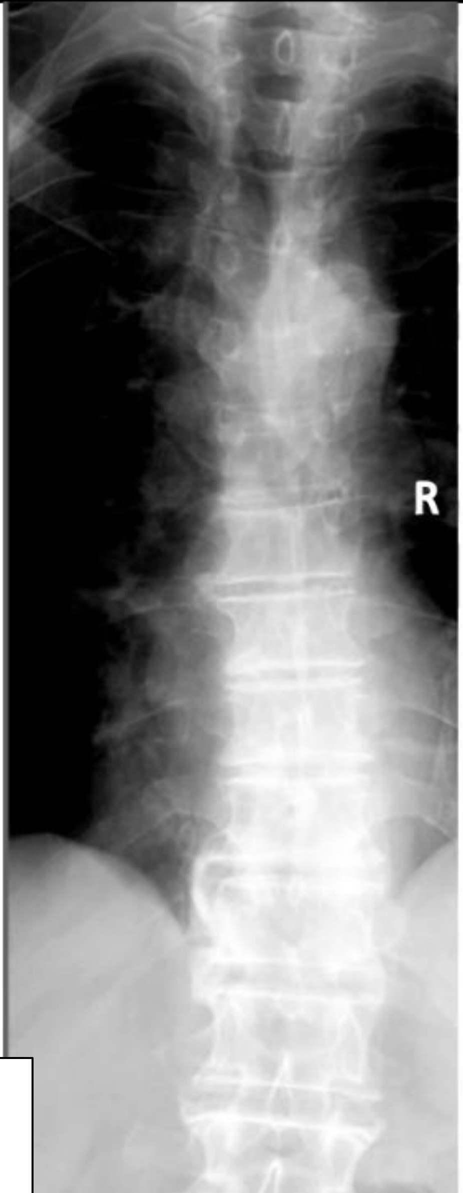

[Old Chronological/Old Pathological/Old Felt Age]

"Especially with this back pain, I'm really starting to feel my age".
